# Physiological role and complex regulation of O_2_-reducing enzymes in the obligate anaerobe *Clostridioides difficile*

**DOI:** 10.1128/mbio.01591-24

**Published:** 2024-08-27

**Authors:** Léo C. Caulat, Aurélie Lotoux, Maria C. Martins, Nicolas Kint, Cyril Anjou, Miguel Teixeira, Filipe Folgosa, Claire Morvan, Isabelle Martin-Verstraete

**Affiliations:** 1 Institut Pasteur, Université de Paris, CNRS UMR6047, Laboratoire Pathogenèse des Bactéries Anaérobies, Paris, France; 2 Instituto de Tecnologia Química e Biológica António Xavier, Universidade Nova de Lisboa, Oeiras, Portugal; 3 Institut Universitaire de France, Paris, France; University of Delaware, Newark, Delaware, USA

**Keywords:** oxygen detoxication, oxygen tolerance, stress response, flavodiiron enzymes, rubrerythrin, Sigma B, Spx, Rex

## Abstract

*Clostridioides difficile*, the major cause of antibiotic-associated diarrhea, is a strict anaerobic, sporulating Firmicutes. However, during its infectious cycle, this anaerobe is exposed to low oxygen (O_2_) tensions, with a longitudinal decreasing gradient along the gastrointestinal tract and a second lateral gradient with higher O_2_ tensions in the vicinity of the cells. A plethora of enzymes involved in oxidative stress detoxication has been identified in *C. difficile*, including four O_2_-reducing enzymes: two flavodiiron proteins (FdpA and FdpF) and two reverse rubrerythrins (revRbr1 and revRbr2). Here, we investigated the role of the four O_2_-reducing enzymes in the tolerance to increasing physiological O_2_ tensions and air. The four enzymes have different, yet overlapping, spectra of activity. revRbr2 is specific to low O_2_ tensions (<0.4%), FdpA to low and intermediate O_2_ tensions (0.4%–1%), revRbr1 has a wider spectrum of activity (0.1%–4%), and finally FdpF is more specific to tensions > 4% and air. These different O_2_ ranges of action partly arise from differences in regulation of expression of the genes encoding those enzymes. Indeed, we showed that *revrbr2* is under the dual control of σ^A^ and σ^B^. We also identified a regulator of the Spx family that plays a role in the induction of *fdp* and *revrbr* genes upon O_2_ exposure. Finally, *fdpF* is regulated by Rex, a regulator sensing the NADH/NAD^+^ ratio. Our results demonstrate that the multiplicity of O_2_-reducing enzymes of *C. difficile* is associated with different roles depending on the environmental conditions, stemming from a complex multi-leveled network of regulation.

**IMPORTANCE:**

The gastrointestinal tract is a hypoxic environment, with the existence of two gradients of O_2_ along the gut, one longitudinal anteroposterior decreasing gradient and one proximodistal increasing from the lumen to the epithelial cells. O_2_ is a major source of stress for an obligate anaerobe such as the enteropathogen *C. difficile*. This bacterium possesses a plethora of enzymes capable of scavenging O_2_ and reducing it to H_2_O. In this work, we identified the role of the four O_2_-reducing enzymes in the tolerance to the physiological O_2_ tensions faced by *C. difficile* during its infectious cycle. These four enzymes have different spectra of action and protect the vegetative cells over a large range of O_2_ tensions. These differences are associated with a distinct regulation of each gene encoding those enzymes. The complex network of regulation is crucial for *C. difficile* to adapt to the various O_2_ tensions encountered during infection.

## INTRODUCTION

*Clostridioides difficile,* formerly known as *Clostridium difficile*, is a Gram-positive sporulating bacterium and a strict anaerobe. *C. difficile* is also an opportunistic pathogen, the first cause of antibiotic-associated diarrhea in developed countries, and the etiological agent of pseudomembranous colitis (1). Spores, which are the form of resistance and dissemination of *C. difficile*, are transmitted through the orofecal route. Following ingestion, spores can germinate in the gastrointestinal tract (GIT) (2). *C. difficile* colonization of the GIT is favored by a dysbiosis of the microbiota often associated with antibiotic treatments (3–5). Indeed, this dysbiosis modifies the pools of metabolites available in the gut including a diminution of the pool of secondary bile acids, which are toxic for *C. difficile* vegetative cells, and an increase in the concentration of primary bile acids, favoring germination (1, 4). Changes in the metabolite pools further allow the colonization of the GIT by the vegetative cells and the production of toxins, which modify the cytoskeleton of enterocytes and disrupt the intestinal barrier (6). Toxin production then triggers an important local inflammation associated with diarrheal symptoms (1, 2).

During its infectious cycle, *C. difficile* is exposed to inflammation-induced oxidative and nitrosative stresses, as well as to low O_2_ tensions, a major stress for this obligate anaerobe. Indeed, there is a decreasing longitudinal gradient of O_2_ along the GIT ranging from 4% to 5% in the small intestine, where spores germinate, to 0.1% to 0.4% in the lumen of the colon (7). In the colon, there is a second lateral O_2_ gradient increasing from the lumen toward the mucus (1%–2%) and the tissues (5%). The O_2_ tensions in the GIT increase during microbiota dysbiosis, which are conditions favoring *C. difficile* infection (8). Indeed, dysbiosis is often associated with a decrease in butyrate-producing commensal bacteria. In physiological conditions, the oxidation of butyrate by the colonocytes consumes O_2_, maintaining hypoxia (9, 10), whereas, in a dysbiotic gut, the depletion of butyrate leads to a switch of colonocytes metabolism to glucose fermentation, decreasing O_2_ consumption (11). This rerouting of epithelial cell metabolism thus favors an increase in O_2_ tensions in the colon (8, 12). *C. difficile*, which encounters O_2_ during the infection, possesses a variety of protection mechanisms and especially detoxication enzymes. In anaerobic bacteria, “Flavodiiron proteins” (Fdp) and rubrerythrins/reverse-rubrerythrins (Rbr/revRbr) play a major role in O_2_ and reactive oxygen species (ROS), namely hydrogen peroxide (H_2_O_2_), detoxication (13). Rbr and revRbr contain both a “diiron” in a four-helix scaffold and a rubredoxin-like (Rd) domain, with an inverted organization between the two families. These proteins act mainly as peroxidases, converting H_2_O_2_ to H_2_O, but they were already reported to also harbor an O_2_-reductase activity, although lower than hydrogen peroxide (14, 15). The Fdp proteins, which act either as NO- or O_2_-reductases (16–18), are a modular family of proteins that share a minimal dimeric form in which each monomer is built by a metallo-β-lactamase-like domain, harboring the catalytic diiron center, and a flavodoxin-like domain containing an FMN (16, 17). Class A Fdps only have this common core, whereas more complex classes have extra domains. For example, class F enzymes have a Rd and a NADH:Rd oxidoreductase (NROR) domain allowing the direct transfer of electrons from NADH to its substrates. Although *C. difficile* is an obligate anaerobe, some strains can grow in the presence of up to 2% O_2_ (19). This rather high O_2_ tolerance can be explained, at least partly, by the presence of four O_2_-reducing enzymes: two Fdp proteins, a Class A (FdpA, CD1157) and a Class F (FdpF, CD1623), and two revRbr, revRbr1 (CD1474) and revRbr2 (CD1524) (15, 18). All four purified enzymes have O_2_-reductase activity *in vitro* (15, 18), and for this reason, will be called O_2_-reductases thereon. While FdpF is a standalone enzyme, receiving electrons directly from NADH, FdpA, and both revRbrs cannot directly take electrons from NADH and therefore need partners that remain to be identified in *C. difficile* (13, 16, 17). The role of these O_2_-reductases in the tolerance to low O_2_ tensions (<0.4%) has been studied (15). At these tensions, a *C. difficile fdpF* mutant is not affected for growth and a *fdpA* mutant has a reduced growth at 0.4% O_2_. A double *revrbr* mutant is almost unable to grow at 0.1 or 0.4% O_2_ but neither single mutant has a phenotype at these O_2_ tensions. A functional redundancy is thus observed for revRbr1 and revRbr2, which share 95% amino acid identity at the protein level (15). Moreover, the genes encoding the four O_2_-reductases are controlled by the alternative sigma factor σ^B^, which is the sigma factor of the general stress response in *C. difficile* (15, 20, 21). The σ^B^ regulon of *C. difficile* also includes many other genes involved in responses to various stress stimuli. Accordingly, a *sigB* mutant is more sensitive to many stresses including low O_2_ tensions but also oxidative and nitrosative stresses (20). In the absence of stress, σ^B^ is sequestered by the anti-sigma factor RsbW, preventing the transcription of its regulon. However, upon stress exposure, the phosphatase RsbZ dephosphorylates the anti-anti-sigma factor RsbV, which then competes with σ^B^ for binding to RsbW, thus relieving the σ^B^ protein (22). The signal sensed and its receptor still remain to be identified.

Identifying a global expression signature of *C. difficile* upon O_2_ exposure is challenging due to the different strains and growth conditions used (19, 23–26). The transcriptomic studies have shown various and sometimes conflicting results suggesting the existence of a rather complex adaptive response of *C. difficile* to O_2_ exposure (19, 24–26). Many genes found differentially regulated upon O_2_ exposure are involved in *C. difficile* metabolism, suggesting the existence of various complex O_2_-dependent rerouting of *C. difficile* metabolism. Some regulators, such as CodY, are known to link metabolic control with stress. Indeed, CodY is inactivated upon metabolic starvation and will thus allow the expression of genes involved both in metabolic rerouting in the stationary phase and toxin production and sporulation. It is worth noting that neither the genes encoding the four O_2_-reductases nor other members of the σ^B^ regulon were found to be differentially regulated upon exposure to hypoxia (8 h at 1.5% O_2_, 4 h at 2% O_2_ or 15 and 60 min at 5% O_2_) (19, 24, 26). However, after a short air exposure to strain 630, the expression of *fdp* and *revrbr* genes is upregulated along with about 100 other genes, including several σ^B^ targets (25). This suggests that those enzymes might be more important for short-term exposure to air than for adaptation to hypoxia.

In this work, we aim to further characterize the physiological role of the O_2_-reductases in the presence of intermediate to high O_2_ tensions, as well as during air exposure. We were able to show that the four O_2_-reductases have different yet overlapping spectra of activity. To better understand the origin of those different spectra of activity, we tested the induction of *fdp* and *revrbr* gene expression in the presence of various O_2_ tensions and identified the different regulators controlling their expression. Each gene encoding an O_2_-reductase seems to have a specific regulation. Indeed, while all genes are transcribed by σ^B^, *revrbr2* and *fdpA* are also transcribed by σ^A^. Moreover, all those genes are controlled by a newly identified oxygen-sensing regulator, while *fdpF* is specifically regulated by Rex.

## RESULTS

### *C. difficile* tolerance to intermediate O_2_ tensions

**Fig 1:**
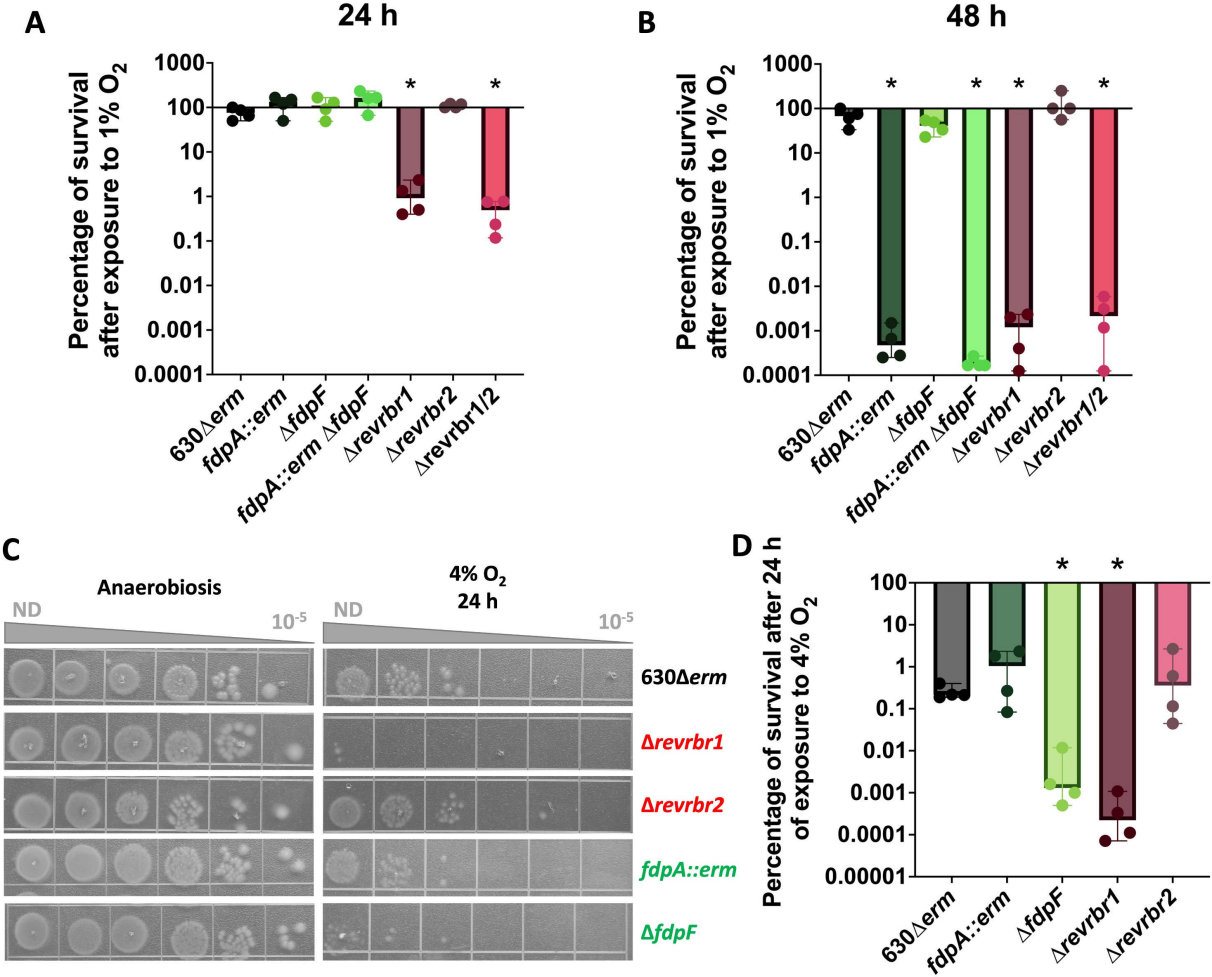
Role of O_2_-reductases in the tolerance to 1% and 4% O_2_. Serial dilutions of the 630∆*erm* strain and single or double mutants inactivated for *revrbr* or *fdp* genes were spotted on TY Tau plates. Plates were incubated either in anaerobiosis for 24 h, in the presence of 1% O_2_ for 24 h or 48 h (Fig. S1A and B) or in the presence of 4% O_2_ for 24 h. Following 24 h incubation in the presence of O_2_, plates were subsequently incubated in anaerobiosis for 24 h. CFU were determined for each experiment. The percentage of survival after exposure to 1% O_2_ relative to the survival in anaerobiosis was plotted at 24 h (**A**) and 48 h (**B**). Pictures are representative of four independent experiments at 4% O_2_ (**C**). The percentage of survival after exposure to 4% O_2_ relative to survival in anaerobiosis was plotted (**D**). Four biological replicates were performed per experiment. For all plots, a median with 95% CI is shown. Mann-Whitney statistical tests were performed. All comparisons were made with the corresponding 630∆*erm* strain. *: *P*-value < 0.05.

The physiological role of the four O_2_-reductases of *C. difficile* in the presence of 0.1%– 0.4% O_2_ tensions has already been studied (15). However, their contribution to the tolerance of *C. difficile* to 1% O_2_, a tension found near the mucus layer and the epithelial cells in the colon that vegetative cells reached during infection (27, 28), remains to be determined. As both triple and quadruple mutants are unable to grow at 0.4% O_2_ (15), we focused our analysis on single and double mutants inactivated for the *fdp* or the *revrbr* genes. We incubated these mutants on agar plates for 24 or 48 h in the presence of 1% O_2_ or anaerobiosis (Fig. 1A and B; Fig. S1A and B). No significant loss of survival was observed for the 630∆*erm* strain after 48 h of growth at 1% O_2_ compared to anaerobiosis (Fig. 1B; Fig. S1A). By contrast, we observed an almost complete loss of survival of the *fdpA::erm* and the double *fdp* mutant after 48 h of exposure to 1% O_2_ (Fig. 1B; Fig. S1A). However, these strains were not affected after a 24-h exposure (Fig. 1A; Fig. S1A). In addition, the phenotype of the ∆*fdpF* mutant remained rather similar to that of the wild-type (WT) strain even after 48 h (Fig. 1A and B; Fig. S1A).

For the double mutant inactivated for both *revrbrs*, we observed a 2-log reduction after 24 h of exposure to 1% O_2_ and a more drastic loss of survival (6-logs) after 48 h (Fig. 1A and B; Fig. S1B). This is in agreement with the drastic phenotype observed at 0.4% O_2_ for this mutant (15). We then tested the phenotype of the single *revrbr* mutants in the same conditions. Upon exposure to 1% O_2_, the ∆*revrbr1* mutant had a phenotype similar to that of the double *revrbr* mutant, both at 24 and 48 h. Surprisingly, the survival of the ∆*revrbr2* mutant was not affected even at 48 h (Fig. 1A and B; Fig. S1B). The difference between both single *revrbr* mutants is striking as the two proteins share 95% amino acid identity and have similar O_2_ reduction activities *in vitro* (15). The revRbr1 seems to play a more important role in the tolerance to 1% O_2_ than revRbr2. After 24 h of growth, the phenotype of the single ∆*revrbr1* and double mutants were, respectively, fully and partially complemented with a *revrbr1* copy carried by a plasmid (Fig. S1C). The *revrbr2* gene expressed under the control of its promoter on a plasmid also fully or partially restored the survival at 24 h of the single ∆*revrbr1* and double *revrbr* mutants (Fig. S1C). The high similarity of both enzymes allows cross-complementation for compensations, even though the role of revRbr2 seems to be minor at 1% O_2_ and is visible only when revRbr1 is absent and when *revrbr2* is overexpressed. Finally, it is interesting to note that the survival of the ∆*revrbr1* mutant is more affected than that of the *fdpA::erm* mutant upon exposure to 1% O_2_ for 24 h (Fig. 1A and B; Fig. S1A and B), suggesting that revRbr1 is more important than FdpA in the tolerance to 1% O_2_. All these results suggest that the tolerance to 1% O_2_ is mainly due to revRbr1 and to a lesser extent to FdpA, whereas FdpF and revRbr2 do not seem to be involved.

### *C. difficile* tolerance to high O_2_ tensions

We then studied the role of the four O_2_-reductases at 4% O_2_, which is physiologically encountered in the small intestine where spores germinate (29). After 24 h of incubation at 4% O_2_, the 630∆*erm* strain had lost around 3 logs of survival, as also observed for the ∆*revrbr2* and the *fdpA::erm* mutants (Fig. 1C and D). However, a significantly more important loss of survival was obtained in the ∆*revrbr1* mutant compared to the WT strain (Fig. 1C and D). This phenotype was fully complemented by expressing the *revrbr1* gene on a plasmid under the control of its promoter (Fig. S1D), whereas the complementation of the ∆*revrbr1* mutant by the *revrbr2* gene was only partial (Fig. S1D). It is worth noting that we observed for the first time a more important loss of survival of the ∆*fdpF* mutant compared with the parental strain (Fig. 1C and D). However, the phenotype of the mutant is not complemented by the introduction of a plasmid-borne copy of the *fdpF* gene with its promoter (Fig. S1D). Altogether, these results suggest that revRbr1 and FdpF contribute to the tolerance of *C. difficile* to high O_2_ tensions, whereas FdpA and revRbr2 seem to play more marginal roles.

### *C. difficile* tolerance to air

**Fig 2:**
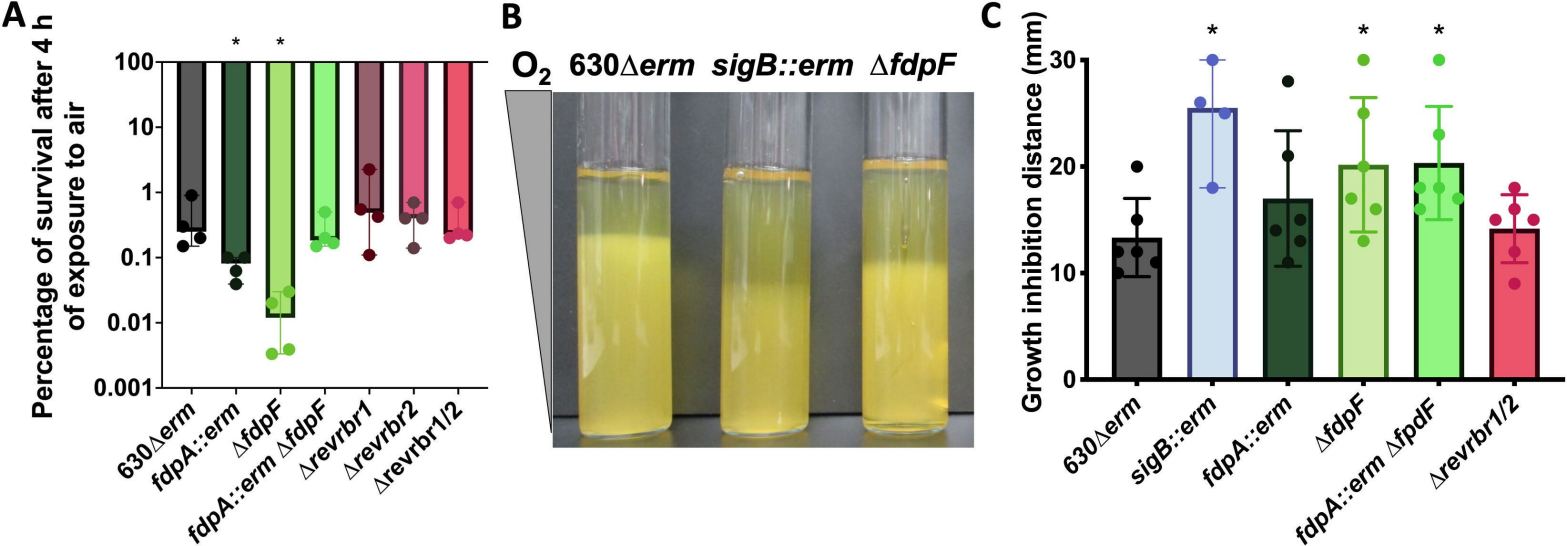
Role of O_2_-reductases in the tolerance to air. Serial dilutions of the 630∆*erm* and the mutants were spotted on TY Tau plates. Plates were incubated either in anaerobiosis for 24 h, or in the presence of air for 4 h (Fig. S2A). Plates incubated in air were subsequently incubated in anaerobiosis for 24 h. CFUs were determined for each experiment and the percentage of survival after exposure to air relative to the survival in anaerobiosis was plotted (**A**). Soft agar tubes were inoculated with various strains and incubated in air (**B**). The growth inhibition distance for each replicate was determined and plotted (**C**). At least four biological replicates were performed per experiment. For all plots, a median with 95% CI is shown. Mann-Whitney statistical tests were performed. All comparisons were made with the 630∆*erm* strain. *: *P*-value < 0.05.

We also investigated *C. difficile* tolerance to air, a high non-physiological O_2_ tension (21%), to test the ability of *C. difficile* to survive harsher conditions at least transiently. Since *C. difficile* is not able to grow in air, we thus exposed transiently vegetative cells to air on plates (4 h) before incubating the plates back for 24 h in anaerobiosis. Only the cells that survived were then able to form colonies. We observed a loss of survival around 3 logs for the 630∆*erm* after 4 h of air exposure (Fig. 2A; Fig. S2A). This result confirmed that air exposure is toxic for *C. difficile*, but that strain 630∆*erm* can still survive a longer air exposure than expected, as previously published (30, 31). After 4 h under air exposure, the survival of the two single *revrbr* mutants and the double mutant inactivated for both *revrbr* was not affected compared to the 630∆*erm* strain (Fig. 2A; Fig. S2A). For the single *fdpA::erm* mutant, we observed a slight but significant decrease in survival compared to the parental strain (1 log) (Fig. 2A; Fig. S2A). Interestingly, the ∆*fdpF* mutant also showed a more drastic survival defect compared to the parental 630∆*erm* strain after 4 h in air (Fig. 2A; Fig. S2A). This phenotype was complemented when we expressed, in this mutant, the *fdpF* gene under the control of its promoter (Fig. S2B). We confirmed the phenotype of the ∆*fdpF* mutant following inoculation of soft agar tubes. After incubation in air, the growth inhibition length reflects the air sensitivity of the strain. As observed for the *sigB::erm* mutant used as a control (20), the zone of growth inhibition for the ∆*fdpF* mutant or for the double *fdp* mutant was significantly longer than that of the parental strain (Fig. 2B and C). This phenotype was complemented by the reintroduction of a copy of *fdpF* on a plasmid (Fig. S2C). By contrast, we did not observe any differential phenotype between the 630∆*erm* and either the *fdpA::erm* mutant or the double *revrbr* mutant (Fig. 2B and C). These results suggest that FdpF is the main O_2_-reductase in air.

### Contribution of the Fdps and revRbrs to O_2_ reduction in crude extracts

**Fig 3:**
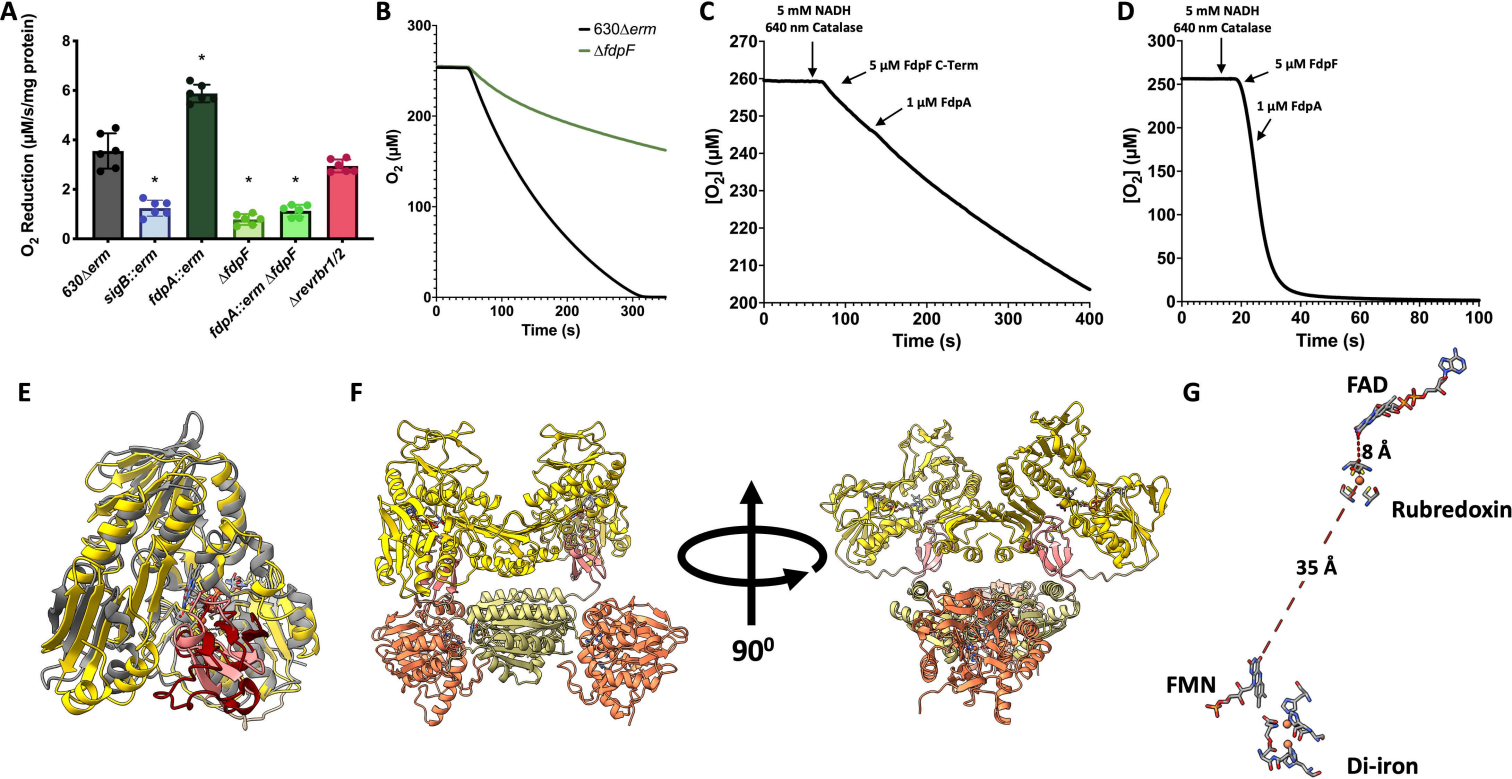
Contribution of FdpA and FdpF to O_2_ reduction in crude extracts and the impossibility of electron transfer between FdpA and FdpF. O_2_-reductase activity was measured in crude extracts of various mutants. The O_2_-reduction capacity is expressed in µM/s/mg protein. Six assays per strain from three independent biological replicates of each strain were performed. Mean and standard deviation are represented. ANOVA tests were performed with a confidence interval of 95% between the mutant strains and the 630∆*erm* strain. *: *P*-value < 0.05 (**A**). O_2_ consumption of crude extracts of the 630∆*erm* strain and the ∆*fdpF* mutant was measured over time. A representative replicate for each strain is shown (**B**). O_2_-reduction assays were performed to assess whether the C-terminal (C-Term) domain of FdpF (**C**) or the full FdpF protein (**D**) could transfer electrons to FdpA. Assays were performed in 50 mM Tris-HCl, pH 7.5 containing 18% glycerol, at 25°C in a buffer equilibrated in air (≈250 µM of O_2_), in the presence of catalase (640 nM, from bovine liver). Assays were initiated by the addition of 5 µM of either FdpF C-Term or FdpF and then 1 µM of FdpA. Assays were performed at least in triplicate and a representative assay is shown. Superposition of the Alphafold3 model of Rd (pink) and NROR (yellow) domains of FdpF (residues 409–843) with the crystallographic structure of *P. aeruginosa* NADH:Rd oxidoreductase complex (PDB 2V3B, gray and dark red) (32) (**E**). AlphaFold3 model structure of FdpF dimer, showing the four domains of each monomer (β-lactamase-like in orange, flavodoxin-like in light yellow, Rd in pink and NROR in yellow) (**F**). Distance between the FAD from the NROR domain, iron from the Rd domain, and FMN from the flavodoxin-like domain predictor from the FdpF AlphaFold3 model (**G**).

To evaluate the contribution of each of these enzymes to O_2_ elimination, we measured *in vitro* in a buffer equilibrated in air the O_2_-reducing activities of crude extracts prepared from anaerobically grown 630∆*erm* and the mutant strains inactivated for the genes encoding the different O_2_-reductases. First, an important loss of O_2_-reductase activity was observed in the *sigB::erm* mutant compared to the 630∆*erm* strain (Fig. 3A), in agreement with the key role of σ^B^ in the control of the expression of the genes involved in O_2_ reduction (15). For the double *revrbr* mutant, the O_2_-reductase activity was similar to that of the parental strain (Fig. 3A). For the single ∆*fdpF* mutant and the double *fdp* mutant, we detected a significant decrease of O_2_-reductase activity comparable to that observed for the *sigB::erm* mutant (Fig. 3A and B). This result indicates that FdpF has the highest ability to reduce O_2_ and cements the importance of FdpF for an efficient O_2_ reduction. In a cellular context, the O_2_-reduction activity of FdpF is more significant than the one from the other enzymes in agreement with the previously reported *in vitro* assays using the purified enzymes (15, 18). It is intriguing to observe a significant increase of the O_2_-reductase activity (+ 60%) in the *fdpA::erm* mutant compared to the WT strain (Fig. 3A). This could be explained by the existence of some compensation at the genetic level between the genes encoding the other O_2_-reductases. To test this hypothesis, we performed RT-qPCR on RNA extracted from the *fdpA::erm* mutant grown in the conditions used for crude extract preparation, that is, after 16 h of growth in anaerobiosis. We showed that neither the *revrbr* genes nor the *fdpF* gene were differentially expressed in the *fdpA::erm* mutant compared to the 630∆*erm* strain (Fig. S3A) suggesting another mechanism involved in this increased activity. Another explanation could be that, in the WT strain, FdpA would hijack electrons from FdpF, that is, electrons would transfer directly from the NROR and Rd domains of FdpF to FdpA (Fig. S3B). We thus tested whether electron transfer can occur between FdpA and either the C-terminal domains of FdpF (NROR and Rd) or the full FdpF protein. We observed no differences in the O_2_ consumption after the addition of FdpA to a medium containing either the C-terminal domain of FdpF or the full FdpF protein (Fig. 3C and D), strongly suggesting that this electron transfer is not efficient or possible. The Alphafold3 model structure of the FdpF dimer is presented in Fig. 3 (Fig. 3E and F) and corroborates this observation (see below). This model predicts a relative position of the Rd and NROR domains on its C-terminal part similar (r.m.s.d. of ~1.2 Å) to what was observed in the complex between the *Pseudomonas aeruginosa* Rd and its NROR (PDB 2V3B) (32). A very short distance of ~8.2 Å is also predicted between the FAD of NROR and the iron from Rd, indicating a direct electron transfer between these two domains and then to the FMN domain (Fig. 3G). These points were demonstrated *in vitro* for both FdpF and FdpH (class H FDP) where mutations in the Rd domain broke the intramolecular electron transfer pathway, leading to a considerable or even total abolishment of their activity (33). In addition, the relative position of the C-terminal and the N-terminal (diiron and FMN domains) parts, with a distance of ~35 Å between the Rd iron and the FMN (Fig. 3F and G), should impair the presence of FdpA in a proper orientation to receive the electrons from the Rd domain of FdpF in agreement with our *in vitro* data (Fig. 3C and D). Finally, we tested the reduction of FdpA with soluble extracts of either the 630∆*erm* strain or the *sigB::erm* mutant. This experiment was done under anaerobic conditions to avoid reoxidation of the enzyme. FdpA can be completely reduced in the presence of both soluble crude extracts and NADH, albeit slowly (Fig. S3D and E). These results thus indicate that the gene encoding the FdpA electron donor is not expressed under σ^B^ control, also supporting ruling out FdpF. This electron donor is, most probably, a so far unidentified NADH oxidoreductase.

### Role of PerR in the control of genes encoding O_2_-reductases

In *Clostridium acetobutylicum,* a *perR* mutant is aerotolerant and PerR strongly represses the expression of the genes encoding the Fdps and the revRbrs, all being induced upon air exposure (34, 35). In *C. difficile,* PerR negatively controls the expression of the *rbr1* operon involved in oxidative stress response (31). PerR senses H_2_O_2_ but might also detect O_2_ as proposed for the PerR regulators of *Staphylococcus aureus* and *Bacillus subtilis* (36, 37). The 630∆*erm* strain harbors a point mutation (T41A) in the *perR* gene making it non-functional and leading to an increased air tolerance (31). So, we wanted to determine whether PerR could still be involved in the regulation of the O_2_-reductase genes in *C. difficile*. To do so, we performed RT-qPCR both on the 630∆*erm* strain and a 630∆*erm* strain harboring a chromosomic *perR*_WT_ allele. We did not observe a significant differential regulation of the O_2_-reductase encoding genes in these two strains (Fig. S4A). Moreover, the *perR*_WT_ strain shows a similar phenotype to the *perR*_T41A_ strain in the presence of 1% O_2_ (Fig. S4B). These results suggest that PerR is not involved in the regulation of O_2_-reductase genes in *C. difficile*.

### *C. difficile* O_2_-reductase gene expression at various O_2_ tensions

**Fig 4:**
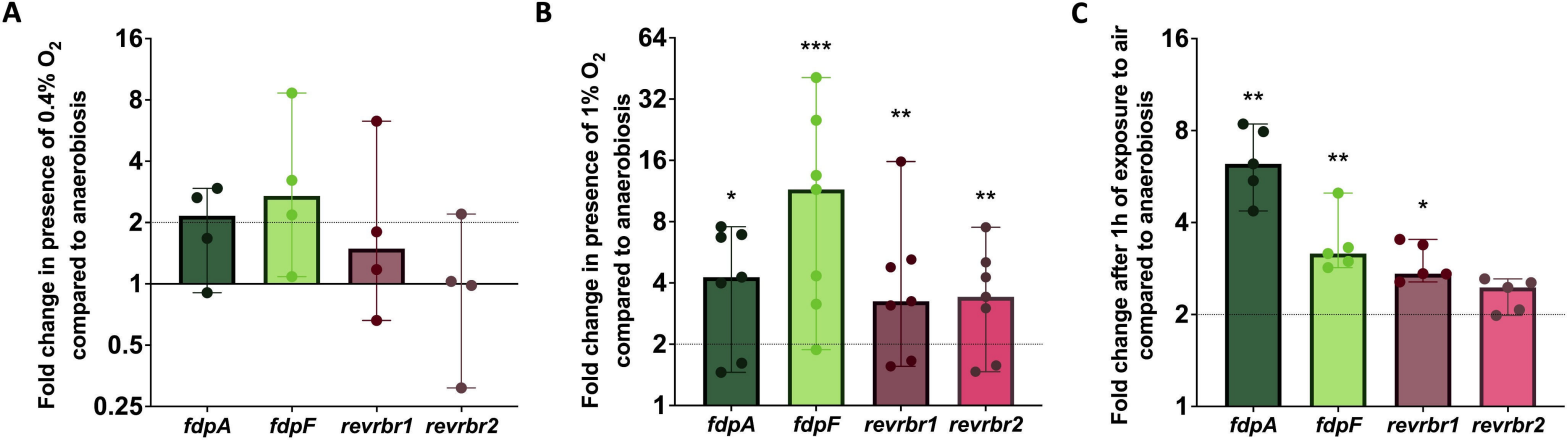
Expression of the genes encoding the O_2_-reductases in the presence of various O_2_ tensions. The expression of the genes encoding the four O_2_-reductases was evaluated by RT-qPCR in the 630∆*erm* strain exposed to different O_2_ tensions [0.4% O_2_ (**A**), 1% O_2_ (**B**) or air (**C**)] and compared with their expression in anaerobiosis. For 0.4% and 1% O_2_, cells were grown for 48 h on agar plates. For air, mid-exponential growing cells were exposed for 1 h to air in six-well plates at 37°C. For all experiments, the control condition was cells grown in anaerobiosis. *gyrA* was used as a reference gene. For all plots, a median with 95% CI is shown. Mann-Whitney statistical tests were performed between the ∆Ct of both conditions compared. *: *P*-value < 0.05; **<0.01; ***<0.001.

The diversity of the role of the O_2_-reductases in the presence of various O_2_ tensions could, at least partly, stem from differences in the expression of the genes encoding these enzymes. We first tested the expression of the four O_2_-reductase genes in the presence of increasing O_2_ tensions. RNAs were sampled in the conditions of our phenotypical assays, that is, a long-term exposure on agar plates. At 0.4% O_2_, we did not observe any differences in expression compared to anaerobiosis (Fig. 4A). By contrast, a significant induction of expression of the four genes (*revrbr1*, *revrbr2*, *fdpA*, and *fdpF*) was shown at 1% O_2_ (Fig. 4B). Testing the exposure to higher O_2_ tensions in similar conditions was more complex, as cells are only able to survive but not to grow at 4% O_2_ and in air. Thus, we performed a transient air exposure in six-well plates. After 1 h of air exposure, we also observed a significant induction of the expression of the genes encoding the O_2_-reductases, except *revrbr2*, which only showed a slight non-significant increase (Fig. 4C). These results show that intermediate O_2_ tensions and air are sensed by *C. difficile* and trigger an O_2_ detoxication response.

### The *revrbr2* gene is expressed under the dual control of a σ^A^- and a σ^B^-dependent promoter

**Fig 5:**
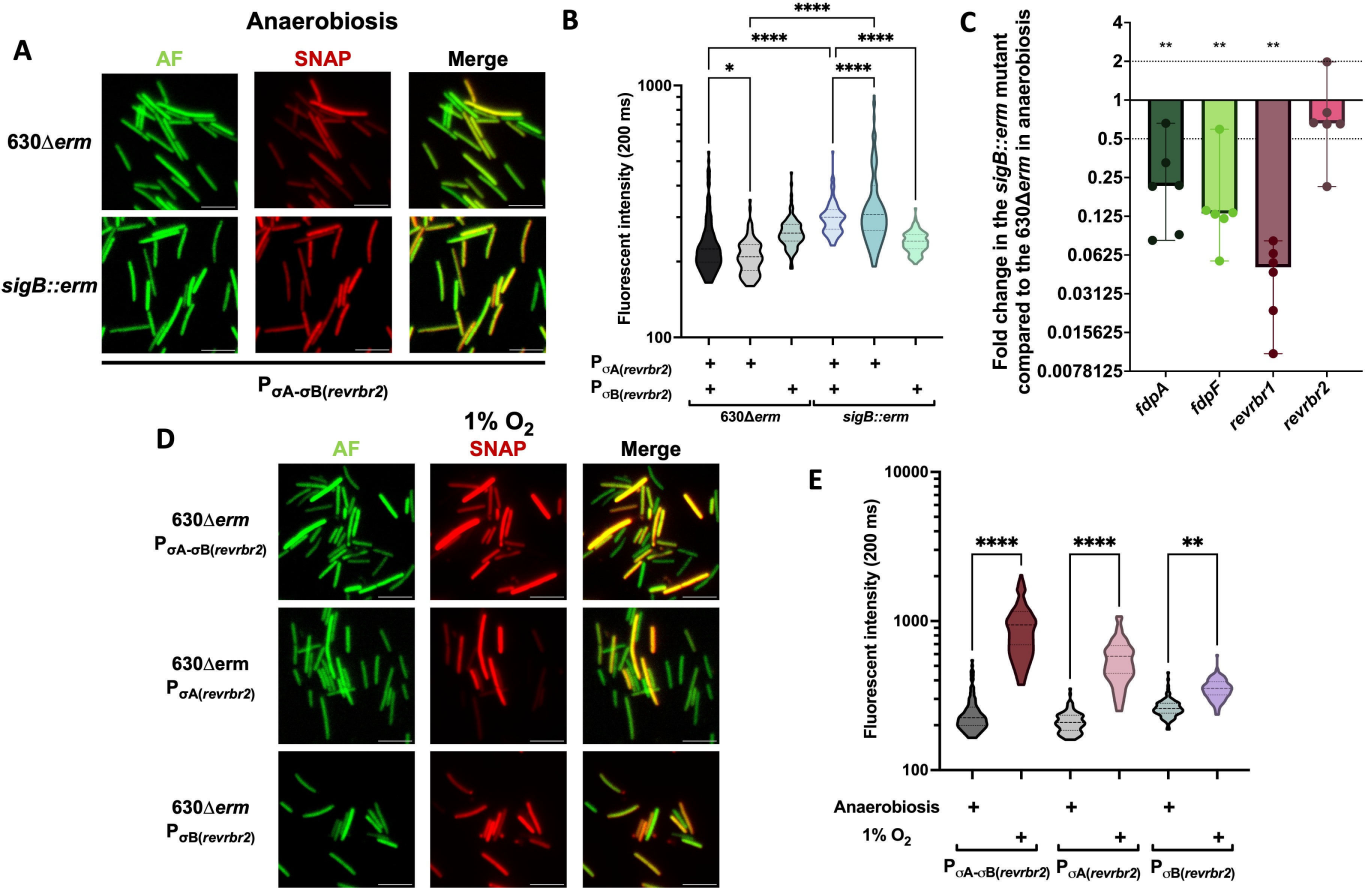
σ^A^- and σ^B^-dependent control of the *revrbr2* gene expression in anaerobiosis and in the presence of O_2_. The expression of the *revrbr2* gene was evaluated using a transcriptional fusion between the promoter region P_σA-σB(_*_revrbr2_*_)_ and the *SNAP^Cd^* fluorescent reporter gene. Constructions containing either only the P_σA(_*_revrbr2_*_)_ or the P_σB(_*_revrbr2_*_)_ were also used. All fusions were transferred to the 630∆*erm* and the *sigB::erm* mutant. Bacteria were cultured for 48 h on agar plates in anaerobiosis. Autofluorescence (AF), SNAP fluorescence, and merge are shown (**A**). Quantification of the fluorescence intensity of the SNAP reporter of images was performed (**B**). 600 cells out of 2 independent experiments were quantified. RT-qPCR experiments were performed using RNA extracted from the 630∆*erm* and the *sigB::erm* strains grown for 48 h on agar plates in anaerobiosis (**C**). Fold changes of expression in the *sigB::erm* mutant compared to the 630∆*erm* strain were plotted. Medians with 95% CI are shown. Six biological replicates were performed per experiment. The different fusions in the 630∆*erm* background were also exposed for 48 h on agar plates to 1% O_2_. Autofluorescence (AF), SNAP fluorescence, and merge are shown (**D**). Quantification of the fluorescence intensity of the SNAP reporter of images was performed (**E**). 600 cells out of 2 independent experiments were quantified. For RT-qPCR, Mann-Whitney statistical tests were performed, while one-way ANOVA were performed for the fluorescence intensity. *: *P*-value < 0.05; **<0.01, ***<0.001, ****<0.0001.

Even if the two revRbr enzymes share 95% of identity at the protein level, different phenotypes for the single *revrbr* mutants were observed. Differences in the expression of the *revrbr* genes might contribute to the distinct role of revRbr1 in the tolerance to intermediate and high O_2_ tensions. Both genes have been previously shown to be controlled by σ^B^ (15, 20). However, the genome-wide transcriptional start site mapping indicated the presence of a second putative σ^A^-dependent promoter upstream of the *revrbr2* gene (Fig. S5A), whereas a second promoter seems to be absent upstream of the *revrbr1* gene (38). This could explain a differential regulation. To confirm the existence of dual regulation of *revrbr2*, we grew the strain carrying a P_σA-σB(_*_revrbr2_*_)_-*SNAP^Cd^* fusion on agar plates for 48 h in anaerobiosis. We were still able to detect fluorescence in the *sigB::erm* mutant (Fig. 5A) and the intensity of fluorescence even slightly increased in this mutant compared to the 630∆*erm* strain (Fig. 5B). In addition, in RT-qPCR experiments, the expression of the *revrbr2* gene was not significantly downregulated in the *sigB::erm* mutant after growth on plates in anaerobiosis (Fig. 5C). These results confirm that the expression of *revrbr2* is not strictly σ^B^-dependent. To analyze more precisely the dual control of the r*evrbr2* gene, we removed either the σ^A^- or the σ^B^-dependent promoter in the P_σA-σB(_*_revrbr2_*_)_-*SNAP^Cd^* fusion (Fig. S5A). A fluorescence was detected for all the constructions in the WT and *sigB::erm* backgrounds (Fig. 5B; Fig. S5B). These results indicate that the σ^A^-dependent promoter also plays a role in the expression of the *revrbr2* gene on plates.

We also compared the expression of the *revrbr1, fdpF* and *fdpA* genes by RT-qPCR in the WT strain and the *sigB::erm* mutant grown on plates. We showed that the expression of the *revrbr1*, *fdpA,* and *fdpF* genes decreased in the *sigB::erm* compared to the WT strain (Fig. 5C). When using transcriptional fusions, we also showed that the fluorescence of the P_(_*_revrbr1_*_)_-*SNAP^Cd^* and the P_(_*_fdpF_*_)_-*SNAP^Cd^* fusion was undetectable in a *sigB::erm* mutant during growth on plates (Fig. S5C and D). This shows that the transcription of the *revrbr1* gene is strictly σ^B^-dependent, while the regulation of the *revrbr2* gene is more complex, as the gene is expressed under the dual control of σ^A^ and σ^B^. The differential physiological role of each revRbr could, at least partly, be explained by these differences of expression.

We then wanted to determine the possible role of σ^B^, the sigma factor of the general stress response (20, 21), in the induction upon long-term exposure to 1% O_2_ of the genes encoding the O_2_-reductases. To test this hypothesis, we used the P_σA-σB(*revrbr2*)_-*SNAP^Cd^*, the P_σA(*revrbr2*)_-*SNAP^Cd^*, and the P_σB(*revrbr2*)_-*SNAP^Cd^* fusions. In the 630∆*erm* strain, we first observed a significant increase in the fluorescence intensity of the P_σA-σB(*revrbr2*)_-*SNAP^Cd^* fusion when cells were grown on plates in the presence of 1% O_2_ (Fig. 5A, D, and E), confirming the induction of *revrbr2* expression at this O_2_ tension. Using the construction containing only the σ^B^ or the σ^A^ promoter, we still observed an increase in the fluorescence intensity in hypoxia (Fig. 5D and E). These results indicate that the induction by O_2_ of the *revrbr2* expression is not exclusively σ^B^-dependent.

### OseR, a new O_2_-sensing repressor, regulates all genes encoding O_2_-reductases

**Fig 6:**
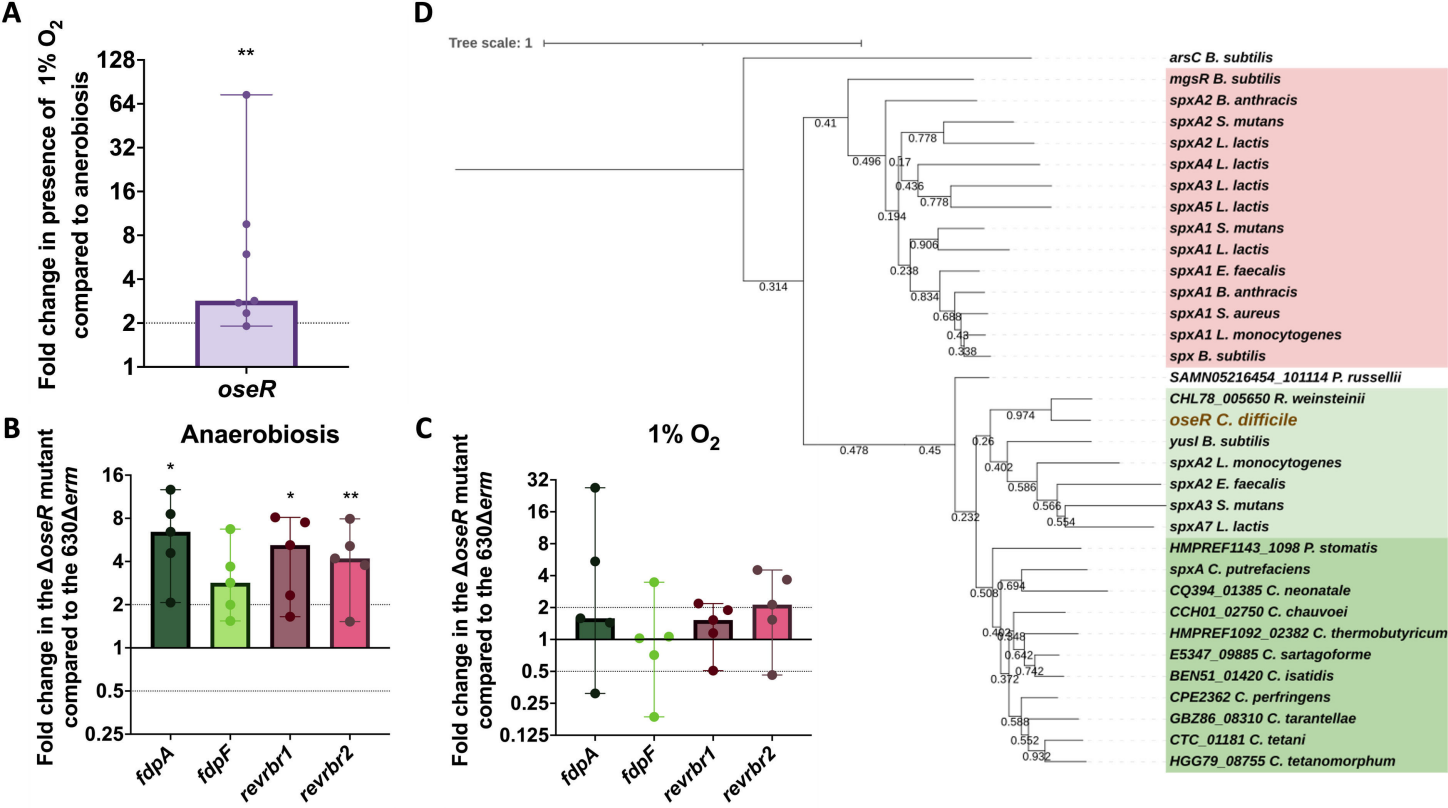
The Spx-like regulator, OseR, regulates the genes encoding the O_2_-reductase in response to O_2_. The expression of the *oseR* gene in the 630∆*erm* strain exposed for 48 h to 1% O_2_ and in anaerobiosis was compared by RT-qPCR (**A**). The expression of the genes encoding the four O_2_-reductases was measured by RT-qPCR in the ∆*oseR* and the 630∆*erm* strain grown for 48 h on agar plates in anaerobiosis or at 1% O_2_. Fold changes correspond to the ratio between the RNA extracted from cells of the ∆*oseR* mutant and the 630∆*erm* strain in anaerobiosis (**B**) or at 1% O_2_ (**C**). Medians with 95% CI are shown. Five biological replicates were performed for each experiment. The sequence of known Spx and Spx-type proteins of Firmicutes was aligned with the sequence of OseR of *C. difficile* and a dendogram was constructed (**D**). Two sub-families are visible: highlighted in red, the canonical Spx and highlighted in green are Spx-like proteins of the YusI subfamily, including OseR (in brown) with two sub-families: in light green, the YusI-like subfamily and in dark green the other proteins of the YusI subfamily found in Clostridia. Mann-Whitney statistical tests were performed between the ∆Ct of the two conditions compared. *: *P*-value < 0.05; ** <0.01.

Since σ^B^ does not seem to be the main O_2_-sensing factor controlling *revrbr* and *fdp* genes, we thus looked for another regulator likely involved in the adaptation to O_2_, which would sense either directly O_2_ or the redox status of the cells. In *Streptococcus mutans*, the *nox* gene encoding the NADH oxidase, which reduces O_2_ to form H_2_O and protects the bacterium from ROS, is induced in the presence of O_2_ *via* a regulator of the Spx family, a family of redox sensing transcriptional factors (39–42). Thanks to a BLAST analysis, we proposed the uncharacterized CD1777 protein (renamed OseR for “Oxygen-sensitive Regulator”), which shares a high identity with regulators of the Spx family, as a good candidate to control the expression of the genes encoding the O_2_-reductases in *C. difficile*. Using 5′-RACE, we mapped a σ^B^-dependent promoter upstream of the *oseR* gene (Fig. S6A) that is in agreement with the 10-fold downregulation of *oseR* expression in the transcriptome of the *sigB::erm* mutant (20, 21). The expression of the *oseR* gene is thus controlled by σ^B^. We also showed that the expression of *oseR* was increased upon long-term exposure to 1% O_2_ (Fig. 6A). Then, we investigated whether OseR could be the regulator involved in the O_2_-dependent induction of *fdp* and *revrbr* genes. We compared by RT-qPCR the impact of *oseR* inactivation on the expression of the genes encoding the O_2_-reductases after growth of the 630∆*erm* or ∆*oseR* mutant in anaerobiosis or the presence of 1% O_2_. In anaerobiosis, we observed a derepression of *fdpA*, *fdpF*, *revrbr1,* and *revrbr2* genes in the ∆*oseR* mutant compared to the 630∆*erm* strain (Fig. 6B), whereas this upregulation disappeared in the presence of 1% O_2_ (Fig. 6C). These results confirm that OseR is involved in the O_2_-dependent control of the expression of the *revrbr* and *fdp* genes. OseR most likely acts as a repressor of these genes in anaerobiosis, whereas O_2_ exposure releases the repression. In accordance, the ∆*oseR* mutant did not show any loss of survival compared to 630∆*erm* when exposed to 1% O_2_ for 48 h (Fig. S6C).

As many Firmicutes encode several proteins of the Spx family (40), we wanted to know whether OseR was a canonical Spx or a Spx-like protein. We thus compared the sequences of OseR to those of proteins of the Spx family in diverse Firmicutes and constructed the corresponding dendogram (Fig. 6D; Fig. S6B). The first striking feature is that there are two major branches in the dendogram, one with all the canonical Spx and some Spx-like proteins, like MgsR of *B. subtilis* (Fig. 6D, red), and a second branch containing more distant proteins and less well characterized Spx-like proteins, including YusI from *B. subtilis* (Fig. 6D, green). Using AlphaFold, we also performed a superposition of the potential structure of the OseR protein with that of YusI and Spx of *B. subtilis*. This suggested that the structures of OseR and YusI seem to be very similar (Fig. S6F) while an additional α-helix is present in Spx (Fig. S6G). OseR clearly belongs to the YusI subfamily of Spx-type proteins (40).

### *fdpF* is also regulated by Rex

**Fig 7:**
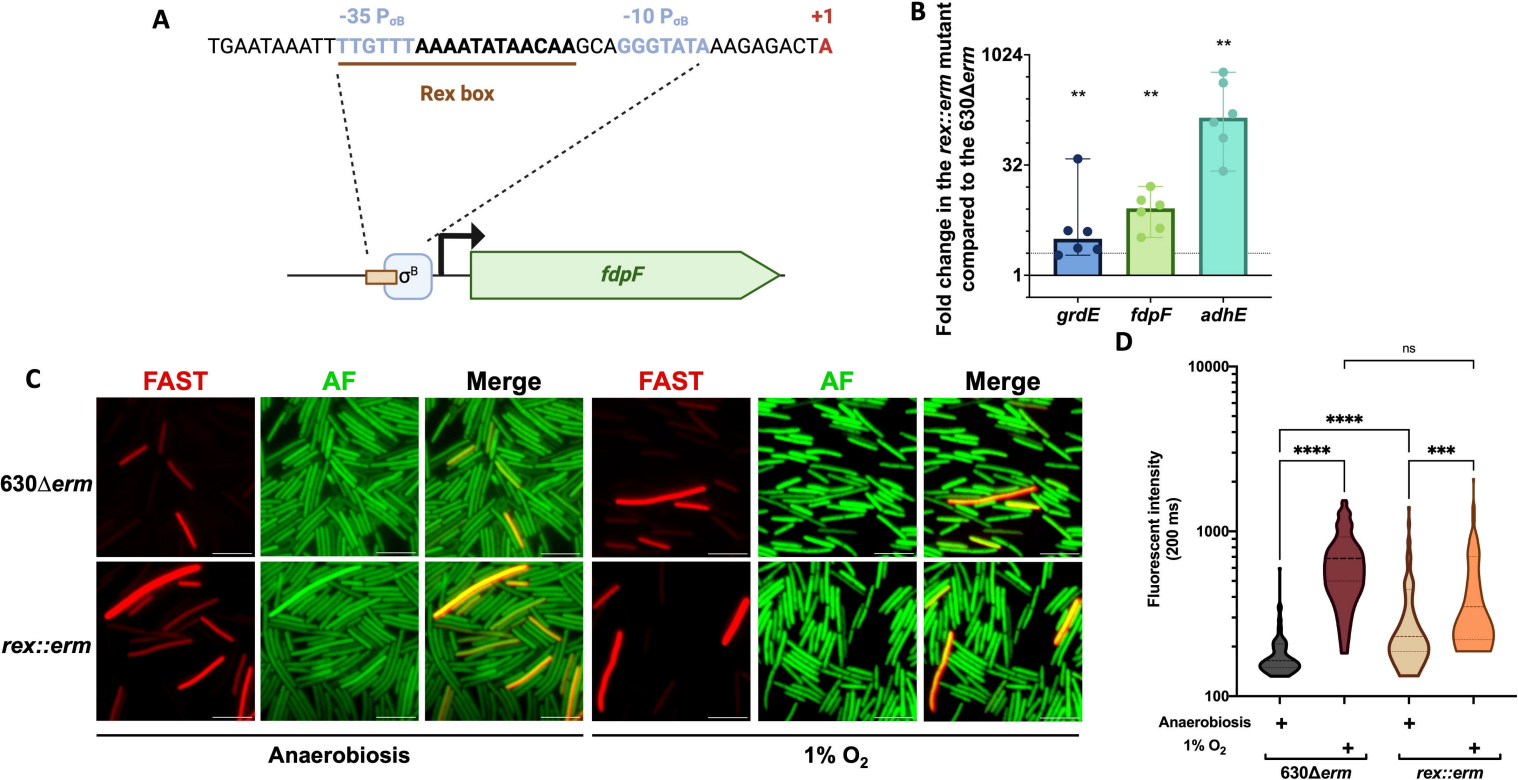
The control of *fdpF* gene expression by Rex. The identified Rex binding box in the promoter region of *fdpF* and the σ^B^-dependent promoter are indicated (**A**). To confirm the regulation of *fdpF* by Rex, RT-qPCR experiments were performed on RNA extracted from the *rex::erm* mutant and the 630∆*erm* strain grown for 48 h on agar plates in anaerobiosis (**B**). Six biological replicates were performed. Medians with 95% CI are shown. Expression of *fdpF* was also tested using a transcriptional fusion between the promoter P_(_*_fdpF_*_)_ and the *SNAP^Cd^* fluorescent reporter gene introduced into the 630∆*erm* strain and the *rex::erm* mutant. Bacteria were grown for 48 h on agar plates in anaerobiosis or at 1% O_2_. Autofluorescence (AF), SNAP fluorescence, and merge are shown (**C**). Quantification of the fluorescence intensity of the SNAP reporter of images in panel C was performed (**D**). 600 bacteria out of 2 independent experiments were quantified. Mann-Whitney statistical tests were performed for qPCR and one-way ANOVA for fluorescence intensity. *: *P*-value < 0.05; **<0.01; ***<0.001; ****<0.0001.

Since FdpF seems to participate more actively under high O_2_ tensions and air, we postulated whether this could be associated with a specific regulation. A putative Rex-binding site overlapping the −35 region of the σ^B^-dependent promoter of *fdpF* (Fig. 7A) has previously been predicted (43), suggesting that Rex might control *fdpF* expression. To test this hypothesis, we compared the expression of *fdpF* in a *rex::erm* mutant and the 630∆*erm* strain. As a control, we also tested known Rex targets such as *adhE* encoding the alcohol dehydrogenase (24, 44, 45) and *grdE* encoding a protein involved in glycine reduction (44, 46, 47). After 48 h of growth on plates, the expression of *adhE* and *grdE* was upregulated as expected (Fig. 7B). The expression of *fdpF* was also significantly upregulated under similar conditions (Fig. 7B). To confirm the regulation of *fdpF* by Rex, we introduced a fusion between the *fdpF* promoter and the *SNAP^Cd^* fluorescent reporter in the 630∆*erm* strain or the *rex::erm* mutant. In anaerobiosis, we were able to show a significant increase in the fluorescence intensity in the mutant compared to the parental strain (Fig. 7C and D), confirming that Rex represses *fdpF* expression in the absence of O_2_. Upon long-term exposure to 1% O_2_, we still observed induction of *fdpF* expression in the *rex::erm* mutant (Fig. 7C and D). In accordance, the *rex::erm* mutant has no differences in survival compared to the 630∆*erm* in the presence of 4% O_2_ and air, conditions where FdpF plays a major role (Fig. S6D and E).

## DISCUSSION

**Fig 8:**
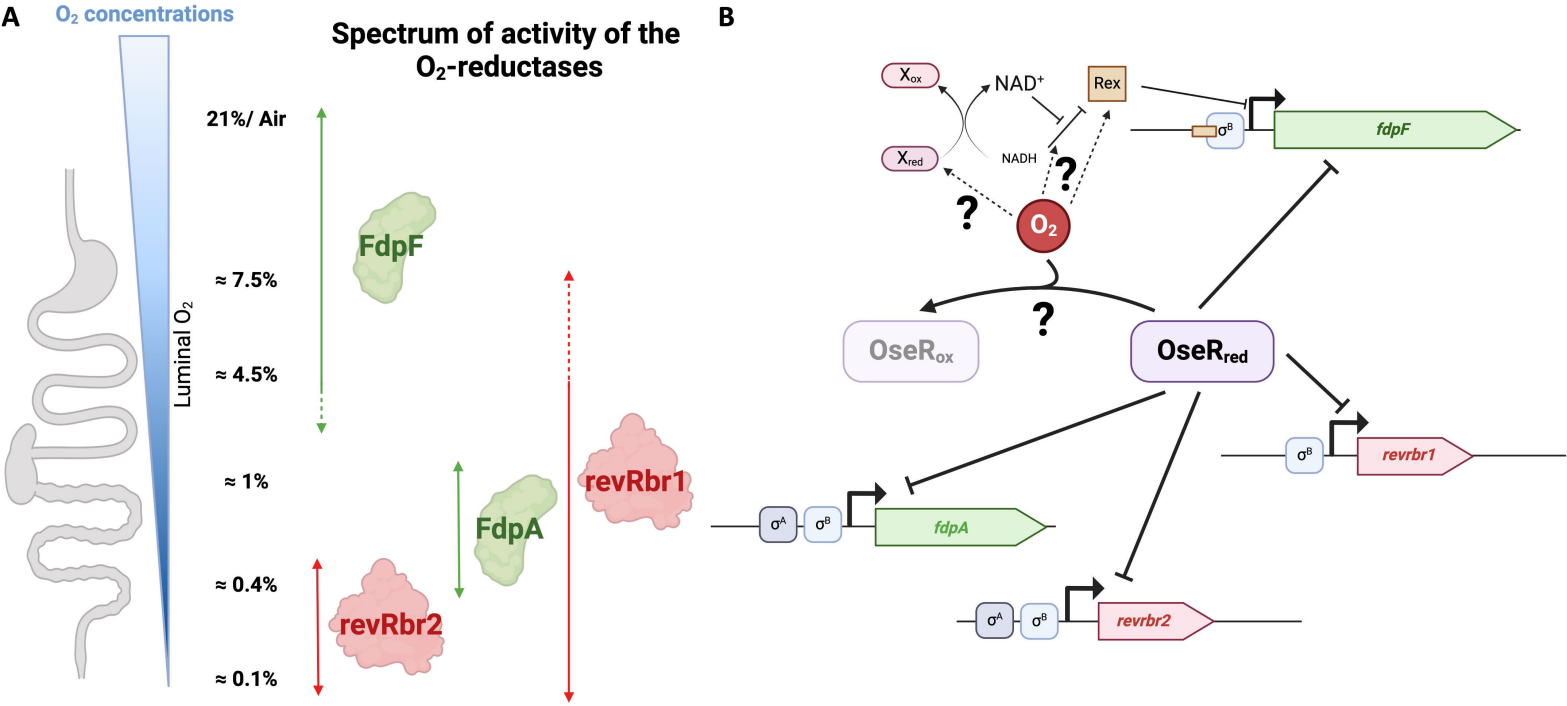
Model of the role and regulation of O_2_-reductases of *C. difficile*. Spectrum of activity of *C. difficile* O_2_-reductases (A). The model is proposed based on the data from the phenotypical assays performed in this work and reference (15). The arrows indicate the activity limits of each O_2_-reductase. The dashed arrows indicate that the spectra of activity might be slightly underestimated since we tested only a limited number of O_2_ tensions. Model of the transcriptional regulation of the genes encoding the four O_2_-reductases (B). The genes *fdpA* and *revrbr2* encoding the two enzymes more specific at the lowest O_2_ tensions are expressed under the control of both σ^B^- and σ^A^-dependent promoters, whereas *fdpF* and *revrbr1* are transcribed only by σ^B^. O_2_ induces the expression of the genes encoding the four enzymes in an OseR-dependent manner. OseR is a repressor under anaerobic conditions but does not repress the genes in the presence of O_2_. Rex represses the expression of the *fdpF* gene in response to the NADH/NAD^+^ ratio.

By combining our data with previous results (15), we showed that the four O_2_-reductases of *C. difficile* have different spectra of activity (Fig. 8A). revRbr2 is indeed more specific to the tolerance to very low O_2_ tensions (<0.4%), whereas FdpA is involved in the tolerance to low and intermediate tensions (0.4%–1% O_2_) and FdpF is important to resist high O_2_ tensions and air (4%–21% O_2_). The spectrum of action of revRbr1 is wider with a role ranging from 0.1% to 4% O_2_. It is worth noting that the two revRbr, sharing more than 95% of homology at the protein level (15), have distinct physiological roles with a more crucial role for the revRbr1 at intermediate to high O_2_ tensions. This is the first study showing a diversity of spectra of activity for O_2_ detoxication enzymes in an anaerobe. In *C. difficile*, the multiplicity of O_2_-reductases with different, yet overlapping spectra of activity, might allow complementary physiological roles covering the large range of the O_2_ tensions encountered during *C. difficile* infection cycle. Tensions from 0.1% to 0.4% O_2_ are found in the lumen of the colon during dysbiosis (48). The role of revRbr and FdpA is thus crucial for the initiation of colonization of the colon. 1% O_2_ is found at the mucus layer and near the epithelial cells that vegetative cells of *C. difficile* reach during infection (27, 28), and O_2_ tensions of 4% are found in the upper GIT, where spores germinate (29). revRbr1 and FdpF, which are important for the tolerance of vegetative cells to 4% O_2_, might play a role in the survival of cells before they attain the colon. It thus seems important for the bacterium to have an arsenal of active protection systems at such O_2_ tensions.

A striking result is the increased O_2_-reduction activity in crude extracts of the *fdpA::erm* mutant compared to those of the 630∆*erm* strain. We showed that this increase does not arise from transcriptional compensation, either through an overexpression of the *fdpF* gene or of the *revrbr* genes in this mutant or from a diminished electron transfer between the C-terminal domain of FdpF and FdpA, that would result in a larger availability of electrons to be used by FdpF. This later hypothesis was not observed *in vitro*, where no electron transfer was detected, and is supported by the model structures predicted for FdpF, that showed an arrangement that should impair a proper docking and electron transfer with FdpA. Electron transfer partners of FdpA and of revRbrs still remain to be identified even if we showed that the genes encoding the partners are not under the control of σ^B^. Several proteins could correspond to NRORs in *C. difficile*, but we can already exclude those controlled by σ^B^ like CD0176. Only two proteins, FdpF and CD0828 (a putative oxidative stress glutamate synthase), contain a Rd domain suggesting that another type of small proteins involved in electron transfer such as flavodoxins or ferredoxins might be involved. Flavodoxins (eight present in *C. difficile*) are of a peculiar interest (49). Indeed, they are less sensitive to oxidative stress, as they rely on an FMN co-factor rather than O_2_-sensitive iron-sulfur clusters found in ferredoxins and would thus be a better electron transfer shuttle upon exposure to O_2_ (50, 51). Interestingly, some genes encoding flavodoxins (*fldX* and *CD2825*) are also induced upon O_2_ exposure in *C. difficile* (26, 49).

All genes encoding O_2_-reductases in *C. difficile* are controlled by the sigma factor of the general stress response, σ^B^ (20, 21). In liquid culture, the inactivation of the *sigB* gene abolished the expression of *revrbr2*, *revrbr1,* and *fdpF* but not of *fdpA*, which is expressed under the dual control of a σ^A^- and a σ^B^-dependent promoter (15). When grown for 48 h on plates, we confirmed that the expression of *fdpF* and *revrbr1* is strictly σ^B^-dependent, but this is not the case for the *revrbr2* gene. Indeed, a *revrbr2*-SNAP fusion remains fluorescent in the *sigB::erm* mutant, due to the existence of a second σ^A^-dependent promoter upstream of *revrbr2* (38). During the late stationary phase on plates, the expression of *revrbr2* seems to rely more on P_σA_ than on P_σB_, while the opposite is observed in liquid culture. Even though σ^B^ plays a role in transcriptional control during the stationary phase (20, 52), a change in the regulation of *revrbr2* can occur during the late stationary phase or on plates. The *revrbr2* gene seems to be regulated by distinct regulatory programs (53, 54). This gene is mainly controlled by σ^B^ during planktonic growth and also by σ^A^ on plates, conditions closer to a microcolony/biofilm-like lifestyle. The differences could arise from a quorum-sensing-dependent control by an uncharacterized regulator or one of the already characterized systems in *C. difficile* (LuxS, RstA, or Agr) (54, 55). We also showed that *revrbr2* expression is induced by O_2_ whether both or just one of the two promoters are present. More importantly, we demonstrated that the induction upon long-term exposure to O_2_ is not exclusively σ^B^-dependent. This has also been previously shown with part of the genes encoding the thioredoxin systems of *C. difficile* under similar conditions (56).

Contrary to *C. acetobutylicum*, the PerR regulator does not seem to be involved in the control of the transcription of genes encoding the O_2_-reductases in *C. difficile*. We identified OseR, which belongs to the Spx family, as a transcriptional factor involved in the regulatory response to O_2_. In other Firmicutes, Spx can act both as a repressor and an activator (39, 57, 58). Spx-dependent activation is mediated *via* an interaction with the α subunit of the RNA polymerase and with promoter regions (59). Spx interacts with a DNA motif (AGCAW_12_AGCG) found upstream of some upregulated genes such as *trxB* of *B. subtilis* and *nox* of *S. mutans* (42, 60, 61). Moreover, upon oxidative stress, the oxidation of the CXXC motif leads to the formation of a disulfide bond, which increases Spx-dependent activation of transcription, by facilitating interactions between the RNA polymerase/Spx complex and the promoter region (59, 62, 63). However, this oxidation is not strictly required for Spx activation (64). OseR, like other clostridial regulators, belongs to the YusI subfamily of proteins rather than to canonical Spx proteins (Fig. 6; Fig. S6). Interestingly, a Spx or YusI homolog could not be identified in *C. acetobutylicum*. Few things are known of *B. subtilis* YusI or its closest relatives in other Firmicutes (40). The YusI subfamily seems to be divided into a group containing most Spx-type regulators found in Clostridia and a group containing YusI-type proteins from non-Clostridial Firmicutes and OseR from *C. difficile*. It is also interesting to note that YusI-type and Spx-type proteins share common motifs: the redox-sensing CXXC and a RPI motif (40, 57). However, all proteins of the YusI subfamily lack the Gly52 residue involved in the interaction of Spx with the α C-terminal domain of the RNA polymerase (57, 65, 66) and nothing is known on the mode of action of those proteins. We showed that OseR seems to act as a repressor of the expression of the *fdp* and *revrbr* genes in anaerobiosis, while this repression is released upon long-term exposure to 1% O_2_. OseR could directly sense O_2_, other sources of oxidative stress, like ROS, or more generally the redox state. Interestingly, in the OseR protein, the CXXC motif (Fig. S6H) is replaced by a CXXV motif (Fig. S6I). An intramolecular disulfide bond, increasing Spx activity in other Firmicutes, cannot be formed in OseR. However, the cysteine residue is exposed at the surface (Fig. S6I) suggesting a possible formation of intermolecular disulfide bonds (65, 67, 68). Residues involved in the interaction of Spx with either σ^A^ or the α C-terminal domain of the RNA polymerase (60) are poorly conserved in OseR, for instance, an important hydrophobic cluster in Spx of *B. subtilis* formed by L46, G52, V71, M74, L76, and L79 is replaced by hydrophilic residues in OseR (Fig. S6J) (60). In addition, neither the DNA motif recognized by Spx (59, 61) nor any other common DNA motif was found upstream of the *revrbr* and *fdp* genes of *C. difficile,* strongly suggesting a different uncharacterized mode of action for OseR. In *B. subtilis*, Spx negatively controls gene expression by a proposed anti-alpha mechanism (57), which could also be a possibility for OseR. Finally, in other Firmicutes, the Spx regulon is quite large and includes many genes involved in stress response (58). In this study, we only focused on the regulation by OseR of the genes encoding O_2_-reductases, but OseR may control other genes involved in oxidative stress response, like systems involved in thiol homeostasis, ROS detoxication, or DNA repair.

We showed that *fdpF* expression is repressed by Rex, a redox regulator that senses the NADH/NAD^+^ ratio (44, 69). However, we did not observe a significant Rex-dependent regulation of *fdpF* after long-term exposure to 1% O_2_ or any differences in survival of a *rex::erm* mutant exposed to increasing O_2_ tensions. In the late stationary phase, the metabolism is slowed down (70) and the NADH/NAD^+^ ratio can be stabilized. *C. difficile* metabolism relies on fermentation for ATP production. Fermentations and Stickland reductive pathways consume NADH leading to NAD^+^ formation. Glycolysis, the incomplete Krebs cycle, transamination reactions, and Rnf regenerate NADH from NAD^+^. As growth is halted during the stationary phase, fermentation pathways are probably slowed leading to a stabilization of the NADH/NAD^+^ ratio. In facultative anaerobes, Rex often controls the hierarchization of the metabolic pathways used by the bacteria. In *Staphylococcus aureus*, Rex acts as a repressor of fermentation pathways (71, 72) in aerobic environments and its target genes are derepressed upon growth in anaerobiosis, where fermentation will be favored (71). Rex also controls genes involved in oxidative stress response. In *S. mutans*, Rex directly regulates the *nox* gene encoding an O_2_ scavenging enzyme (42), whereas in *C. acetobutylicum* Rex regulates indirectly many genes encoding oxidative stress detoxication enzymes, including the *revrbr* and the *fdp* genes (73). In *C. acetobutylicum*, exposure to H_2_O_2_ reduces the NADH/NAD^+^ ratio leading to a repression of the Rex regulon. This control might increase the availability of NADH for detoxication enzymes (73). In *C. difficile*, it is interesting to note that *fdpF* is the sole O_2_-reductase gene directly controlled by Rex (43). In *C. difficile*, Rex represses alternative NAD^+^ regeneration pathways (44), suggesting that FdpF might act as one. Indeed, the standalone enzyme, FdpF, might be a more reliable and faster pathway for NAD^+^ regeneration than the other O_2_-reductases. This would be coherent with the data obtained in crude extracts, suggesting that FdpF is the fastest and most efficient O_2_-reducing enzyme of *C. difficile* and the combination of an increased NAD^+^ regeneration with an efficient O_2_ detoxication could be an advantage for cells exposed to O_2_. Finally, many stress-response-associated enzymes, including the O_2_-reductases, consume NADH for their activity (13). When FdpA and both revRbrs are active upon O_2_ exposure, a decrease in the NADH/NAD^+^ ratio might occur and thus leading to a repression of the Rex regulon, *fdpF* included.

Finally, a model of regulation of the four genes encoding O_2_-reductases in *C. difficile* can be proposed (Fig. 8B). These genes are all expressed under the control of σ^B^, even if *fdpA* and *revrbr2* are also transcribed by σ^A^. It is interesting to note that the expression of *fdpF* and *revrbr1*, encoding the O_2_-reductases involved in the tolerance to higher O_2_ tensions, are strictly dependent on σ^B^, the sigma factor of the general stress response. FdpF and revRbr1 are likely mainly produced under stressful conditions triggering σ^B^ activity (22). By contrast, *fdpA* and *revrbr2* are also transcribed by σ^A^, that ensure a basal level of O_2_ reduction and protection even at very low O_2_ tension. The production of FdpA and revRbr2 might be further enhanced under the control of σ^B^ when the stress increases. Moreover, we showed that all four genes are repressed in anaerobiosis by OseR, a regulator from the Spx/YusI family and that this repression is released upon exposure to O_2_, probably through oxidation of OseR. Finally, *fdpF* is also regulated by Rex, and thus is repressed upon increase of the NADH/NAD^+^ ratio, even though a direct or indirect link with exposure to O_2_ remains to be established. Multiple levels of controls exist. For instance, the *fdpF* gene is dually regulated by OseR and by Rex in addition to its control by σ^B^. Other uncharacterized cross-regulations for *fdpA* and *revrbr* genes yet to be characterized might complete the proposed model in the future (Fig. 8B). A rather more complex regulatory network exists as the *oseR* gene itself is transcribed under the control of σ^B^ and its expression is induced by O_2_ (20, 21). In this paper, we focused on the transcriptional regulation of the O_2_-reductase genes, but we cannot exclude additional levels of control. First, as FdpA and both revRbr require electron transfer partners for their activity, the expression of the genes encoding those partners could also be regulated. Indeed, in *C. acetobutylicum*, the genes encoding the Rd and the NROR are also controlled by Rex (73). Control at post-translational levels could also modulate the activity or the stability of O_2_-reductases. Indeed, even though FdpA and both revRbr seem to have similar O_2_-reductase activity *in vitro* (15), their *in vivo* activity might also depend on the O_2_ tensions. In addition, the O_2_-reductases seem to be phosphorylated, with even FdpF being phosphorylated at multiple sites (74). The effect of phosphorylation on the activity of the Fdp or revRbr enzymes will deserve further investigations. As a conclusion, we started to decipher a complex multi-level network of control of the O_2_-reductases of *C. difficile*. The regulatory network identified strengthen the spectra of activity of the various enzymes and contributes to the different physiological roles of these multiple O_2_-reductases in an obligate anaerobe, exposed to many varying O_2_ tensions during its infectious cycle.

## MATERIALS AND METHODS

### Strains and growth conditions

Both *Escherichia coli* and *C. difficile* strains used in this study are listed in Table S1. *E. coli* strains were grown in LB (tryptone 10 g.L^−1^, yeast extract 5 g.L^−1^, and NaCl 5 g.L^−1^). *C. difficile* strains were grown under anaerobiosis (5% H_2_, 5% CO_2_, 90% N_2_) in TY medium (bacto tryptone 30 g.L^−1^, yeast extract 20 g.L^−1^, pH 7.4), BHI (Brain Heart Infusion 37 g.L^−1^) or a peptone-containing medium Pep-M [proteose peptone no. 2 at 40 g.L^−1^ (BD Diagnostics, USA), Na_2_HPO_4_ 5 g.L^−1^, KH_2_PO_4_ 1 g.L^−1^, NaCl g.L^−1^, MgSO_4_ 0.1 g.L^−1^] (75). All media were solidified by the addition of 15 g.L^−1^ (BHI and TY) or 20 g.L^−1^ (LB) of agar. When necessary, antibiotics were added at the following concentrations: ampicillin (Amp) 100 µg.mL^−1^, chloramphenicol (Cm) 15 µg.mL^−1^, thiamphenicol (Tm) 15 µg.mL^−1^, erythromycin (Erm) 2.5 µg.mL^−1^, cefoxitin (Cfx) 25 µg.mL^−1^, and cycloserine 250 µg.mL^−1^. When needed, Taurocholate (Tau) at 0.05% wt/v was added to favor spore germination.

### Plasmid and strain construction

The clostron gene knockout system (76, 77) was used to inactivate the *rex* gene (*CD0171*), yielding the insertional mutant strain 630Δ*erm rex::erm*. We designed primers to retarget the group II intron of pMTL007 to insert it into the *rex* gene in sense orientation after nucleotide 193 in the coding sequence. The PCR product generated by overlap extension was cloned between the HindIII and BsrG1 sites of pMTL007 to obtain pDIA5908. *C. difficile* transconjugants obtained with *E. coli* HB101(RP4) containing pDIA5908 were selected on BHI agar containing Tm and Cfx and then plated on BHI agar containing Erm. We performed PCRs on the chromosomal DNA of transconjugants to verify the integration of the intron into the *rex* gene. The ∆*oseR* mutant was obtained using the *CD25717.1*-mediated allelic chromosomic exchange method. 1 kb fragments located upstream and downstream of this gene were PCR amplified from 630Δ*erm* genomic DNA using the primer pair CM1-CM2 and CM3-CM4. Purified PCR fragments were then introduced into the pDIA6753 plasmid using a Gibson Assembly master mix (Biolabs). The plasmid obtained, pDIA6864, introduced in HB101(RP4) *E. coli* strain was transferred by conjugation into the *C. difficile* 630Δ*erm* strain. Transconjugants were selected on BHI plates supplemented with Tm and *C. difficile* selective supplement (SR0096; Oxoid). Isolation of faster growing single crossing-over integrants was performed by serial restreaking on BHI plates containing Cfx and Tm. Single-crossover integrants were then restreaked on BHI plates supplemented with anhydrotetracycline (200  ng.mL^−1^), 1% glucose, and 0.1% cysteine, allowing the isolation of double-crossover events. After confirmation of plasmid loss (Tm-sensitive clones), the presence of the expected deletion in clones was checked by PCR using oligonucleotides CM5 and CM6.

### Survival assays in the presence of intermediate/high O_2_ tensions or in air

A culture of *C. difficile* in TY Tau was inoculated at 1:50 from an overnight culture in the same medium. After 3 h of growth (OD_600nm_ between 0.35 and 0.8), we prepared an inoculum at an OD_600nm_ of 0.5 in TY Tau. For each strain, serial dilutions by 10 up to 10^−5^ were prepared. 5 µL of each dilution and the non-diluted inoculum were plated on calibrated square plates containing 28 mL of TY Tau agar to avoid the presence of spores. For all types of assays, a control plate was kept at 37°C in anaerobiosis for 24 h. The other plates were incubated at 37°C either in hypoxia (5% CO_2_, × % O_2_, 95 × % N_2_) or in air (21% O_2_) for various durations: 24 h and 48 h in the presence of 1% O_2_, 24 h in the presence of 4% O_2_ and 4 h in air. Plates exposed to those conditions were subsequently incubated again for 24 h at 37°C in anaerobiosis. Plates incubated for 48 h under hypoxia were not further incubated in anaerobiosis. CFU were numerated after incubations.

The inhibition of growth by air was tested in soft agar tubes (78). Briefly, 20 µL of an overnight culture of *C. difficile* grown anaerobically in the Pep-M medium was mixed with 10 mL of the Pep-M medium containing 0.4% agar at 45°C in a screw cap tube. The tubes were then incubated aerobically at 30°C for 40 h allowing the establishment of an O_2_ gradient. We measured the distance from the top of the agar to the edge of visible bacterial growth, that is, the zone of growth inhibition by air.

### Determination of O_2_-reducing activities in *C. difficile* crude extracts

To prepare crude extracts, an overnight culture of *C. difficile* strains in TY was used to inoculate 10 mL of the same medium (1:50). After 8 h, this culture was diluted (1:100) in 200 mL of TY medium. After 16 h of growth, the OD_600nm_ of the culture was measured and the cells were harvested by centrifugation at 6,000 rpm for 15 min at 4°C. Cells were resuspended in 25 mL of ice-cold sterile PBS, transferred to 50 mL tubes, and centrifugated. The pellets were resuspended in 10 mL of ice-cold sterile PBS and pelleted again by centrifugation for 10 min at 5,000 rpm and 4°C. Pellets were then stored at −20°C. Cells were resuspended in 50 mM Tris-HCl pH 7.5 containing 18% glycerol and disrupted by three cycles in a French-Press apparatus at 16,000 psi (Thermo) in the presence of 1 mg.mL^−1^ DNAse (Applichem). The crude extracts were centrifuged at 25,000 *g* for 30 min and at 138,000 *g* for 1.5 h at 4°C to remove cell debris and membrane fraction, respectively. The soluble fractions divided in aliquots were stored at −20°C.

The O_2_-reducing activity of the soluble fractions was measured amperometrically with a Clark-type electrode selective for O_2_ (Oxygraph-2K, Oroboros Instruments, Innsbruck, Austria). The assays were performed in 50 mM Tris–HCl, pH 7.5 containing 18% glycerol. The O_2_-reducing activity was evaluated at 25°C in an air-equilibrated buffer (≈250 µM of O_2_), in the presence of catalase (640 nM, from the bovine liver) and superoxide dismutase (240 nM, from bovine erythrocytes) to avoid the influence of ROS (superoxide and H_2_O_2_) formed by incomplete O_2_ reduction. The soluble fraction was added to the reaction mixture and the O_2_ concentration was allowed to stabilize (avoiding the variability originated by the different endogenous reductant concentrations). Then, the reaction was initiated by the addition of 5 mM of NADH. The turnover rates (s^−1^) were calculated by subtracting the experimental slope (µM.s^−1^) before and after the addition of NADH and dividing by the total protein amount used in each assay. The total protein concentration of the soluble fractions was determined by the Bradford method using bovine serum albumin as standard (79).

### Electron transfer assays to FdpA

The anaerobic reduction of 30 µM of FdpA was performed in the presence of 60 µg.mL^−1^ and 50 µg.mL^−1^ of the soluble extracts of either 630∆*erm* or the *sigB::erm* mutant and 200 µM of NADH, and monitored by UV-Visible spectroscopy in a Shimadzu UV-1800 spectrophotometer, inside an anaerobic chamber (Coy Lab Products, Grass Lake, MI, USA).

The FdpA O_2_ reduction assays using FdpF C-Term or FdpF as electron donors were performed in 50 mM Tris-HCl, pH 7.5 containing 18% glycerol, at 25°C in an air-equilibrated buffer (≈250 µM of O_2_), in the presence of catalase (640 nM, from the bovine liver). Assays were initiated by the addition of 5 µM of either FdpF C-Term or FpdF and then 1 µM of FdpA.

### Alphafold3 model structure predictions

The FdpF dimer model structure was predicted by Alphafold3 (80), using FdpF amino acid sequence and considering three iron atoms and 1 FAD molecule per protein monomer. The FMN molecules were added by running the PDB file generated by Alfaphold3 with Alphafill algorithm [https://alphafill.eu/ (81)]. Structure superimposition with the Rd and NADH:Rd oxidoreductase complex of *Pseudomonas aeruginosa* (PDB 2V3B), distance measurements, and figures were generated with ChimeraX v1.7.1 (82–84). Model structures of OseR from *C. difficile* and of YusI and Spx of *B. subtilis* were predicted by Alphafold3, using their respective amino acid sequences. Structure superposition was generated with ChimeraX v1.7.1.

### RNA extraction, RT-qPCR, and 5′RACE

To check for compensation at the genetic level and the role of PerR, cells from the 630∆*erm* strain (both the *perR*_T41A_ and *perR*_WT_) and the *fdpA::erm* mutant were harvested after 16 h of growth at 37°C in liquid TY medium. For hypoxia exposition, cells were harvested after 48 h of growth on a TY-calibrated agar plate incubated either in anaerobiosis or in hypoxia (0.4% or 1% O_2_) at 37°C. Cells from agar plates were resuspended in 3 mL of killing buffer (20 mM Tris HCl 7,5; 5 mM MgCl_2_; 20 mM NaN_3_) prior a rapid centrifugation. For air exposition, cells from an exponential phase culture were exposed for 1 h in air at 37°C in six-well plates containing 2 mL of medium per well to maximize the surface/volume ratio and optimize the gas exchange.

The culture pellets were resuspended in RNApro solution (MP Biomedicals) and RNA was extracted using the FastRNA Pro Blue Kit (MP Biomedicals). RNA was then purified using the Direct-Zol RNA Miniprep kit (Zymo). cDNA synthesis and quantitative PCR were performed as previously described (85, 86). Primers used are listed in Table S2. As both revRbrs share more than 95% of identity at the protein level, primers for RT-qPCR were carefully designed in regions that were different in *revrbr1* and *revrbr2* to distinguish each gene. The specificity of the primers was confirmed by RT-qPCR on RNA extracted from either a ∆*revrbr1* or a ∆*revrbr2* single mutant. The *gyrA* gene was used as a reference gene. The relative change in gene expression was determined using the ∆∆Ct approach (87).

To determine the transcription initiation site of *oseR*, the 5′ RACE (Rapid Amplification of cDNA Ends, Invitrogen kit) technique was used. mRNAs were extracted from the 630∆*erm* strain after 5 h of growth in TY. Using SuperScript II reverse transcriptase in the buffer provided [20 mM Tris-HCl (pH 8.4), 50 mM KCl, MgCl_2_ 2.5 mM, dNTP 0.4 mM, DTT 0.01 mM], a single-strand cDNA was synthesized from a site internal to the unknown 5′ end of the *oseR* mRNA using the oligonucleotide IMV694. The matrix mRNA was eliminated by treatment with H and T1 RNAses. A polyC tail was then added to the 3′ ends of the cDNA *via* the terminal deoxynucleotidyl transferase. A PCR was then performed using a primer complementary to the polyC tail (containing an arbitrary sequence in 5′) and IMV753, a primer specific of *oseR*. A second amplification of the obtained PCR product was performed with oligonucleotides specific for the start of *oseR* (IMV1211) and a primer hybridizing with the arbitrary sequence located upstream of the polyC (Abridged Anchor Primer APP). The PCR products were cloned into the plasmid pGEMT easy (Promega). 4 to 8 plasmids corresponding to white colony-forming transformants were extracted and sequenced to determine the start of transcription.

### Transcriptional *SNAP^Cd^* fusions

The transcriptional fusions of the promoter region of *revrbr1*, *revrbr2*, *fdpA,* and *fdpF* with the *SNAP^Cd^* reporter gene using the pFT47 vector have been already published (88, 89). The deletion of the σ^A^ and σ^B^ promoter (Fig. S3A) has been performed by inverse PCR using pDIA6459 (pFT47-P_σA-σB(_*_revrbr2_*_)_-*SNAP^Cd^*) as template and oligonucleotides IMV1588 and IMV1576 or IMV1577 and IMV1578, respectively. This allowed to construct plasmids pDIA7285 (pFT47-P_σA(*revrbr2*)_*-SNAP^Cd^*) and pDIA7290 (pFT47-P_σB(*revrbr2*)_*-SNAP^Cd^*). The plasmids as well as the other SNAP fusions were introduced into *E. coli* HB101(RP4) and then transferred by conjugation into the *C. difficile* 630Δ*erm* strain, the *sigB::erm* or the *rex::erm* mutant.

### Fluorescence microscopy and image analysis

To monitor the expression of the different transcriptional fusions, the strains were grown for 48 h at 37°C on calibrated TY plates either in anaerobiosis or in the presence of 1% O_2_. The plates were initially inoculated using a 4 h culture of the different strains. SNAP labeling and fluorescence microscopy were performed as previously described (89). The images were taken with exposure times of 200 ms for autofluorescence and 300 ms for SNAP. Cells were observed on a Nikon Eclipse TI-E microscope 60× objective and captured with a CoolSNAP HQ2 Camera. For quantification of the SNAP-TMR Star signal resulting from transcriptional fusions, the mean fluorescence intensity of each bacterium was determined using ImageJ. 600 bacteria from two biological replicates were analyzed per condition.

### Bioinformatic analysis

OseR was identified as a Spx family protein using the BLAST tool from the Uniprot website (www.uniprot.org) (90, 91) to BLAST Spx from *B. subtilis* against the database restricted to *C. difficile*. Spx-like regulators in Clostridia were also identified by using the BLAST tool from the Uniprot website to BLAST Spx from *B. subtilis* against the database restricted to Clostridia. Spx and Spx-like regulator sequences from Firmicutes (File S1) were aligned using the MEGA software (version 11.0.13) based on the MUSCLE algorithm (92). A distance tree was then constructed using neighbor-joining method with a JTT substitution model with 500 bootstraps. ArsC from *B. subtilis* (93) was used as an outgroup to root the tree. The final dendrogram was produced using iTOL (94).

### Statistical analysis

For survival assays on a plate or in soft agar tubes, Mann-Whitney tests were performed. For qPCR, Mann-Whitney tests were performed between the ∆Ct of both conditions compared. The results obtained for the different mutant strains were compared with the wild type (630Δ*erm*) ones (at least five assays per strain from three independent biological replicates of each strain) using ANOVA test with a confidence interval of 95%. All data are presented as the mean value ±standard deviation (SD). Finally, for microscopy quantification, one-way ANOVA followed by Tukey’s multiple comparison test was performed. Graphs and statistical analysis were performed using GraphPad Prism (version 10.2.1). Schematics were drawn using Biorender (www.biorender.com).

## SUPPLEMENTAL MATERIAL

Supplemental figures mbio.01591-24-s0001.docx Figures S1 to S6.

Supplemental tables mbio.01591-24-s0002.docx Tables S1 and S2.

ASM does not own the copyrights to Supplemental Material that may be linked to, or accessed through, an article. The authors have granted ASM a non-exclusive, world-wide license to publish the Supplemental Material files. Please contact the corresponding author directly for reuse.
