# Supplementary material for: Physiological role and complex regulation of O2-reducing enzymes in the obligate anaerobe Clostridioides difficile
Source: mBio. 2024 Aug 27;15(10):e01591-24. doi: 10.1128/mbio.01591-24 (PMC11481553; doi:10.1128/mbio.01591-24)
Supplement: Supplemental figures — Figures S1 to S6. [file mbio.01591-24-s0001.docx]

**
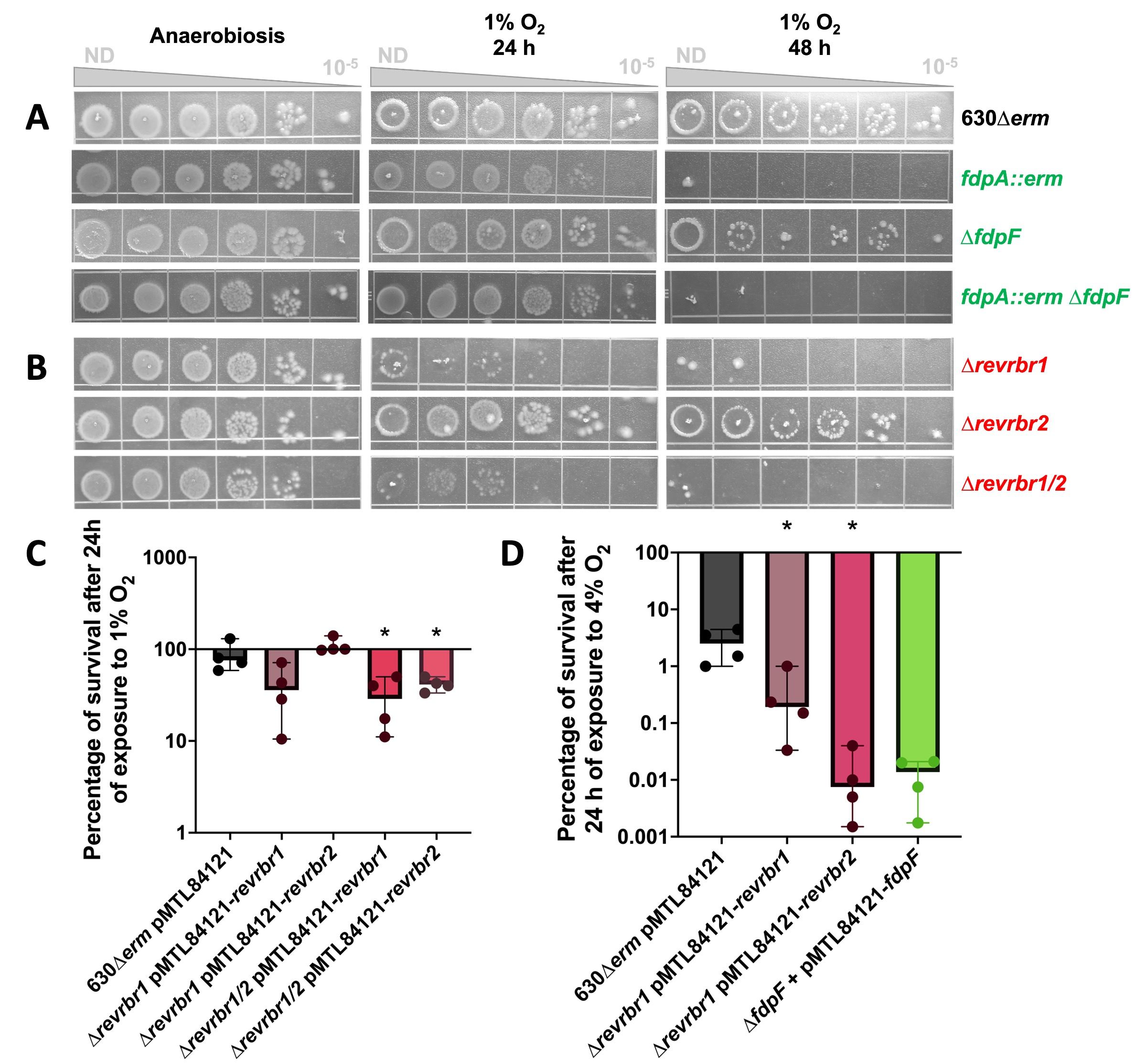
Figure S1:** **Role of O_2_-reductases in the tolerance to intermediate and high O_2_ tensions.**

Serial dilutions of the mutants or complemented strains were spotted on TY Tau plates. Plates were incubated either in anaerobiosis for 24 h, at 1% O_2_ for either 24 h or 48 h (A, B, C) or at 4% O_2_ for 24 h (D). Following the 24 h incubation in the presence of O_2_, plates were subsequently incubated in anaerobiosis for 24 h. Pictures are representative of four independent experiments for the *fdp* mutants (A) or the *revrbr* mutants (B) exposed to 1% O_2_. For the complementation of the *fdpF* mutant (D) or of the single or double *revrbr* mutants (C, D), CFUs were determined and the percentage of survival after 24 h of exposure to 1% O_2_ (C) or to 4% O_2_ (D) relative to the survival in anaerobiosis were plotted. 4 biological replicates were performed per experiment. For all plots, median with 95% CI are shown. Mann-Whitney statistical tests were performed. All comparisons were made with the corresponding 630∆*erm* pMTL84121 strain. *: p-value <0.05.


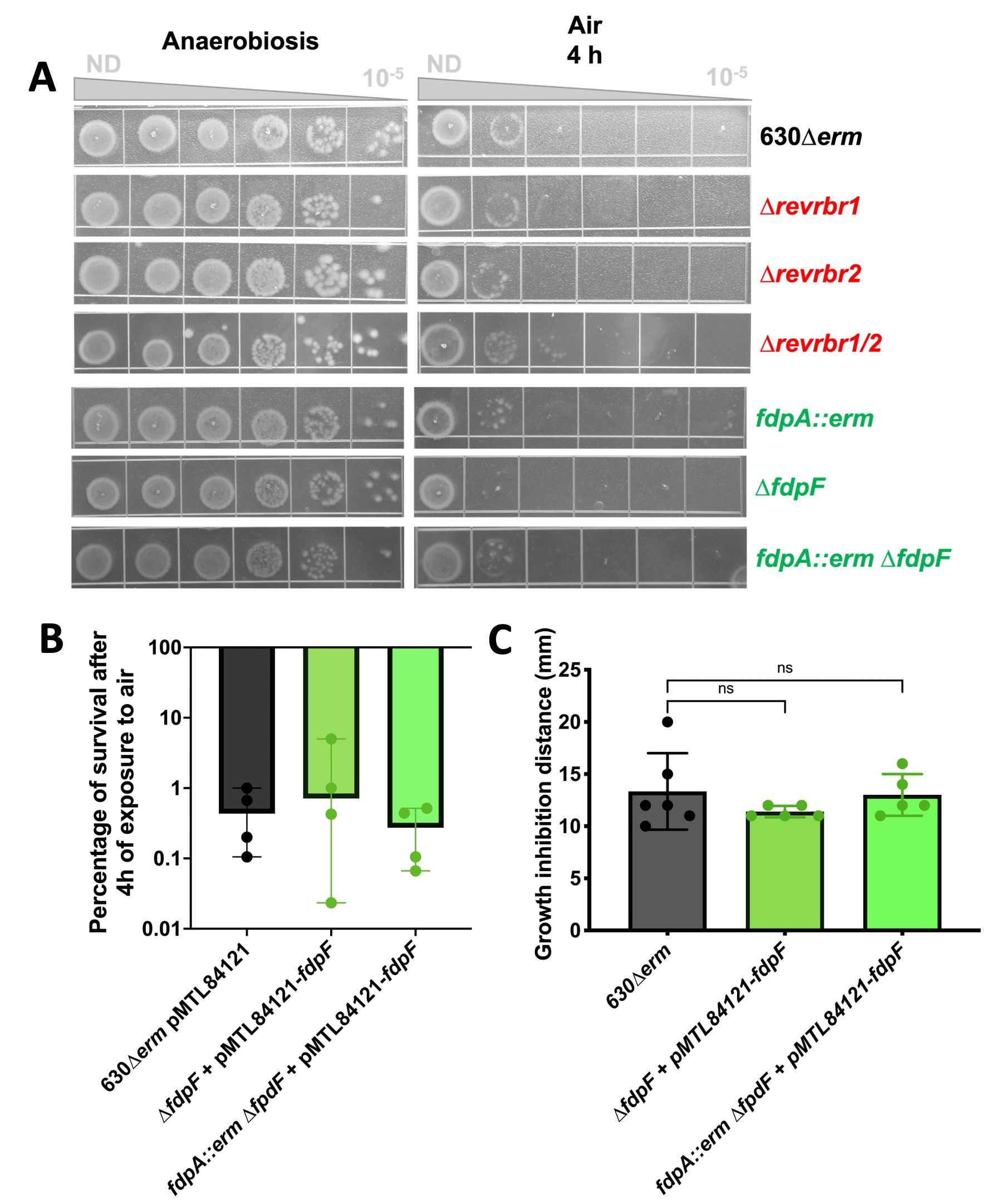


**Figure S2:** **Role of O_2_-reductases in the tolerance to air.**

Serial dilutions of the mutants or complemented strains were spotted on TY Tau plates. Plates were incubated either in anaerobiosis for 24 h or in presence of air for 4 h and subsequently incubated in anaerobiosis for 24 h. Pictures are representative of four independent experiments. For complemented strains, CFUs were determined for each experiment and the percentage of survival after exposure to air relative to the survival in anaerobiosis were plotted (B). Soft agar tubes were inoculated with the various strains and incubated in air. The growth inhibition distance for each replicate of complemented strains was determined and plotted (C). At least 4 biological replicates were performed per experiment. For all plots, median with 95% CI are shown. Mann-Whitney statistical tests were performed. All comparisons were made with the corresponding 630∆*erm* strain.





**Figure S3: O_2_-reductase encoding genes are not differentially regulated in the *fdpA::erm* mutant and the electron transfer partner of FdpA is not controlled by** **σ^B^**

The expression of the *fdpF* and *revrbr* genes was evaluated by qPCR in the *fdpA::erm* strain grown for 16 h and compared to the 630∆*erm*. 4 biological replicates were performed per experiment. Median with 95% CI are shown. Mann-Whitney statistical tests were performed. All comparisons were made with the corresponding 630∆*erm* strain (A). FdpA needs electron transfer partners, as it cannot directly use electrons from NADH (B). Representation of the domains of FdpF. Domains from N-terminal to C-terminal are the metallo-β-lactamase-like domain with the diiron center (Fe-Fe), the flavodoxin domain (FMN), the rubredoxin domain (Rd) and the NADH:Rd oxidoreductase domain (NROR) (C). Usually, electron partners are a NROR that transfer electrons from the NADH to a Rd, that ultimately transfer electrons to FdpA. *C. difficile* FdpA reduction with *C. difficile* WT soluble extract (D) and *C. difficile* *sigB::erm* soluble extract (E) was assessed overtime. Assays were performed with 30 µM of FdpA and 60 or 50 µg.mL^-1^ of cell extracts, respectively. NADH concentration was 200 µM. Insets represent incubation of FdpA and cell extracts, under anaerobic conditions, before addition of NADH, showing a characteristic absorbance band of the oxidized form of FdpA at 450 nm. This band is gradually lost overtime after addition of NADH in presence of the soluble extract, suggesting a reduction of FdpA. As it was previously shown that NADH cannot directly reduce FdpA (15), this implies that the partner required for electron transfer from NADH to FdpA is present in both crude extracts. (D,E): the experiments have been performed at least in triplicates and a representative replicate is shown.


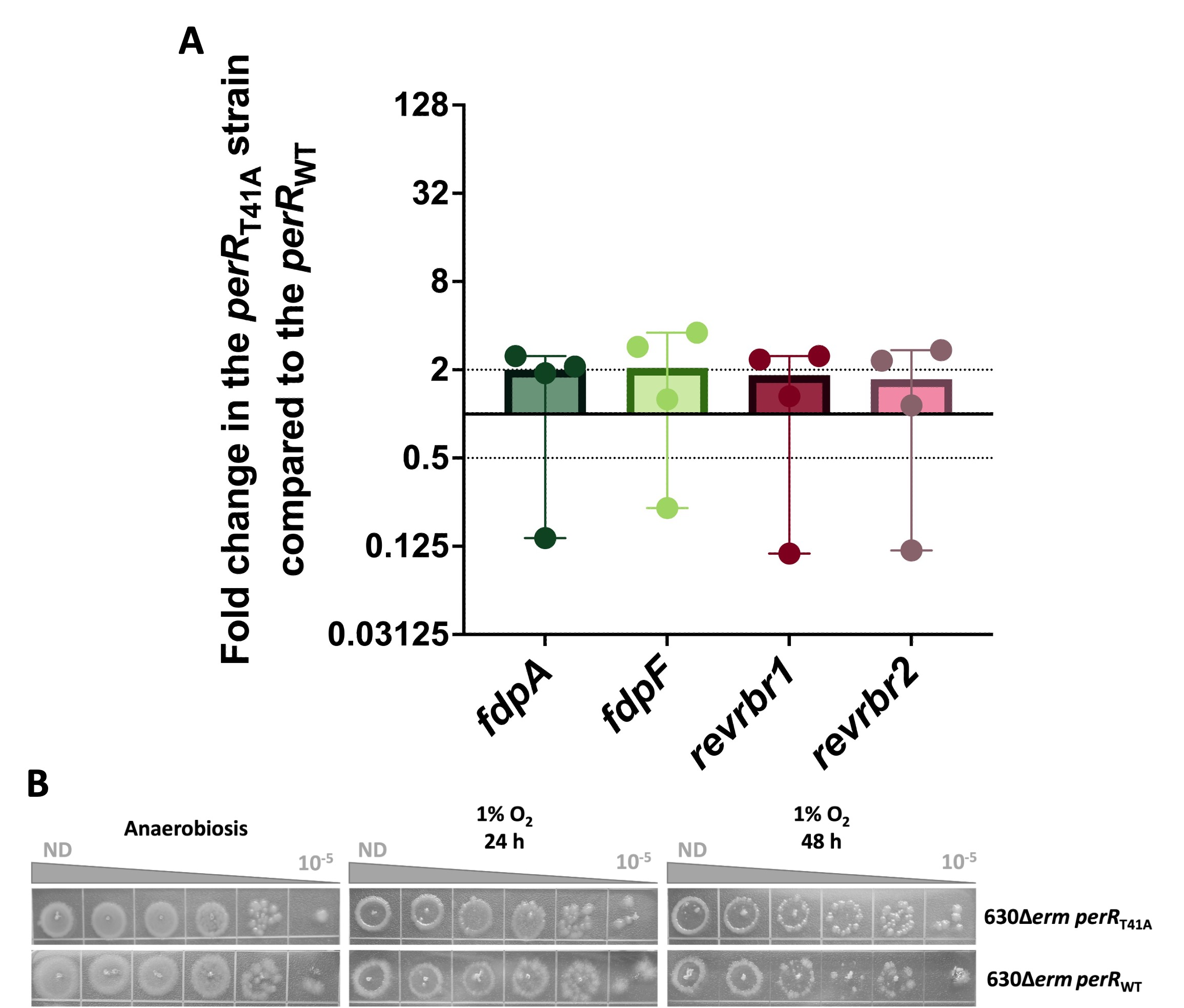


**Figure S4: PerR is not involved in the control of the O_2_-reductases**.

To confirm the absence of regulation of genes encoding the O_2_-reductase by PerR, RT-qPCR experiments were performed on RNA extracted from a 630∆*erm* *perR*_WT_ and the 630∆*erm perR*_T41A_ strain grown overnight in anaerobiosis (A) Serial dilutions of the 630∆*erm* *perR*_WT_ and the 630∆*erm perR*_T41A_ strain were spotted on TY Tau plates. Plates were incubated either in anaerobiosis for 24 h, in the presence of 1% O_2_ for 24 h or 48 h. Following 24 h incubation in the presence of O_2_, plates were subsequently incubated in anaerobiosis for 24 h. Pictures are representative of four independent experiments (B). 4 biological replicates were performed. Medians with 95% CI are shown. Mann-Whitney statistical tests were performed between the ∆Ct of both conditions compared.





**Figure S5:** **Expression of the genes encoding O_2_-reductases in the *sigB::erm* mutant.**

The deletions of P_σA_ or P_σB_ in the constructions corresponding to P_σA(_*_revrbr2_*_)_- or P_σB(_*_revrbr2_*_)_-SNAP fusions are indicated. (A). Constructions containing either only the P_σA(_*_revrbr2_*_)_ or the P_σB(_*_revrbr2_*_)_ were transferred to the 630∆*erm* and the *sigB::erm* mutant. Bacteria were cultured for 48 h on agar plates in anaerobiosis. Autofluorescence (AF), SNAP fluorescence and merge are shown (B). Fusions of either the P_(_*_revrbr1_*_)_ or the P_(_*_fdpF_*_)_ and the *SNAP^Cd^* fluorescent reporter gene were introduced in the 630∆*erm* strain and in the *sigB::erm* mutant. Bacteria were cultured for 48 h on agar plates. Autofluorescence (AF), SNAP fluorescence and the merge were shown for P_(_*_revrbr1_*_)_- SNAP*^Cd^* (B) and P_(_*_fdpF_*_)_- SNAP*^Cd^* (C).





**Figure S6:** ***oseR* promoter, Spx-type protein alignment and phenotypes of mutants inactivated for OseR and Rex.**

(A) The promoter of *oseR* was mapped by 5’RACE and a σ^B^-dependent promoter was identified. (B) The sequence of known Spx and Spx-type proteins of Firmicutes were aligned with the sequence of OseR of *C. difficile*. (C) Common motives of the Spx family were highlighted (in red, the CXXC motif, in orange the G52 residue and in green the RPI motif). Two sub-families are visible: highlighted in red, the canonical Spx and in light green are highlighted proteins of the YusI subfamily, including OseR (in brown). (D, E) Serial dilution of the 630∆*erm*, the ∆*oseR* and the *rex::erm* mutant were spotted on TY Tau plates. Plates were incubated either in anaerobiosis for 24 h, at 1% O_2_ for 48 h (C), at 4% O_2_ for 24 h (D), or in air for 4 h (E). For the 24 h and air incubations, plates were subsequently incubated in anaerobiosis for 24 h. CFUs were determined for each experiment as well as the percentage of survival after exposure to O_2_ relative to the survival in anaerobiosis. 4 biological replicates were performed per experiment. For all plots, median with 95% CI are shown. Mann-Whitney statistical tests were performed. All comparisons were made with the 630∆*erm* strain. (F) Superposition of the AlphaFold3 model structure of OseR (green) and YusI from *B. subtilis* (light blue). The residue D52 of OseR is shown in orange. (G) Superposition of the AlphaFold3 model structure of OseR (green) and Spx from *B. subtilis* (magenta). The residue D52 of OseR is shown in orange. (H,I) Highlight of the Cys residue of Spx (H; cyan) and of OseR (I; dark blue), showing that cysteines are accessible to the solvent. (J) Zoom of the superposition of the AlphaFold3 model structure of OseR (green) and Spx from *B. subtilis* (magenta). Residues of the hydrophobic cluster of Spx are in light blue. The corresponding residues of OseR are in orange.
