# Supplementary material for: Physiological role and complex regulation of O2-reducing enzymes in the obligate anaerobe Clostridioides difficile
Source: mBio. 2024 Aug 27;15(10):e01591-24. doi: 10.1128/mbio.01591-24 (PMC11481553; doi:10.1128/mbio.01591-24)
Supplement: Supplemental tables — Tables S1 and S2. [file mbio.01591-24-s0002.docx]

**Table S1. List of strains and plasmids used in this study**

| **Strains** | **Genotypes** | **Origins** |  |
| --- | --- | --- | --- |
| ***E. coli*** | | | |
| HB101 (RP4) | *supE*44 *aa*14 *galK2 lacY1* ∆(*gpt-proA*) 62 *rpsL20* (Str^R^)*xyl-5 mtl-1 recA13* ∆(*mcrC-mrr*) *hsdS*_B_(r_B_^-^m_B_^-^) RP4 (Tra^+^ IncP Ap^R^ Km^R^ Tc^R^) | Laboratory stock |  |
| NEB10 | Δ(*ara-leu*) *7697 araD139 fhuA* Δ*lacX74 galK16 galE15 e14-* ϕ*80dlacZ*Δ*M15* *recA1 relA1 endA1 nupG rpsL* (Str^R^) *rph spoT1* Δ(*mrr-hsdRMS-mcrBC*) | NewEnglands BioLabs |  |
| ***C. difficile*** | | | |
| 630Δ*erm* | *perR*_T41A_ | Laboratory stock |  |
| 630Δ*erm perR*_WT_ | *perR*_WT_ | Laboratory stock | |
| CDIP546 | 630Δ*erm sigB*::*erm* | Kint *et al*., 2017 |  |
| CDIP697 | 630Δ*erm* Δ*revrbr1* | Kint *et al.*, 2020 |  |
| CDIP1369 | 630Δ*erm* Δ*fdpF* | Kint *et al.*, 2020 |  |
| CDIP691 | 630Δ*erm* Δ*revrbr2* | Kint *et al.*, 2020 |  |
| CDIP588 | 630Δ*erm fdpA*::*erm* | Kint *et al.*, 2020 |  |
| CDIP714 | 630Δ*erm* Δ*revrbr1* Δ*revrbr2* | Kint *et al.*, 2020 |  |
| CDIP1370 | 630Δ*erm* Δ*fdpF fpdA::erm* | Kint *et al.*, 2020 |  |
| CDIP801 | 630Δ*erm* Δ*revrbr1* pMTL84121-P-*revrbr1* | This work |  |
| CDIP840 | 630Δ*erm* Δ*revrbr1* pMTL84121-P-*revrbr2* | This work |  |
| CDIP802 | 630Δ*erm* Δ*revrbr1* Δ*revrbr2* pMTL84121-P-*revrbr1* | Kint *et al.*, 2020 |  |
| CDIP800 | 630Δ*erm* Δ*revrbr1* Δ*revrbr2* pMTL84121-P-*revrbr2* | Kint *et al.*, 2020 |  |
| CDIP1448 | 630Δ*erm* Δ*fdpF* pMTL84121-P-*fdpF* | This work |  |
| CDIP1449 | 630Δ*erm fdpA ::erm* Δ*fdpF* pMTL84121-P-*fdpF* | Kint *et al.*, 2020 |  |
| CDIP5 | 630∆*erm rex::erm* | This work |  |
| CDIP1412 | 630∆*erm* ∆*CD1777* | This work |  |
| CDIP659 | 630∆*erm* pFT47-P_σA-σB(_*_revrbr2_*_)_-*SNAP^Cd^* | Kint *et al.*, 2020 |  |
| CDIP661 | 630∆*erm* pFT47-P_(_*_revrbr1_*_)_-*SNAP^Cd^* | Kint *et al.*, 2019 |  |
| CDIP2206 | 630∆*erm* pFT47-P_σA(_*_revrbr2_*_)_-*SNAP^Cd^* | This work |  |
| CDIP2207 | 630∆*erm* pFT47-P_σB(_*_revrbr2_*_)_-*SNAP^Cd^* | This work |  |
| CDIP658 | 630∆*erm* pFT47-P_(_*_fdpF_*_)_-*SNAP^Cd^* | Kint *et al.*, 2020 |  |
| CDIP668 | 630∆*erm sigB::erm* pFT47-P_(_*_revrbr1_*_)_-*SNAP^Cd^* | Kint *et al.*, 2020 |  |
| CDIP669 | 630∆*erm* *sigB::erm* pFT47-P_(_*_fdpF_*_)_-*SNAP^Cd^* | Kint *et al.*, 2020 |  |
| CDIP670 | 630∆*erm sigB ::erm* pFT47-P_σA-σB(_*_revrbr2_*_)_-*SNAP^Cd^* | Kint *et al.*, 2020 |  |
| CDIP2208 | 630∆*erm sigB ::erm* pFT47-P_σA(_*_revrbr2_*_)_-*SNAP^Cd^* | This work |  |
| CDIP2209 | 630∆*erm sigB ::erm* pFT47-P_σB(_*_revrbr2_*_)_-*SNAP^Cd^* | This work |  |
| CDIP2190 | 630∆*erm rex::erm* pFT47-P_(_*_fdpF_*_)_-*SNAP^Cd^* | This work |  |
| **Plasmids** | **Characteristics** | **Origins** |  |
| pMTL84121 | Replicative plasmid in both *E. coli* and *C. difficile* which can be transferred by conjugation | Heap *et al*. 2009 (76) |  |
| pMSR | Plasmid for gene inactivation by ACE in *C. difficile* | Laboratory stock |  |
| pFT47 | *SNAP^Cd^*, Cm^R^-Tm^R^ | Pereira *et al*. 2013 (83) |  |
| pMTL007 | Clostron vector, CmR-TmR | Heap *et al*. 2007 (77) |  |
| pDIA6864 | pMSR ACE ∆*CD1777* | This work |  |
| pDIA5908 | pMTL007-*rex::erm* 193s | This work |  |
| pDIA6457 | pFT47-P_(_*_revrbr1_*_)_-*SNAP^Cd^* | Kint *et al.*, 2019 |  |
| pDIA6459 | pFT47-P_σA-σB(_*_revrbr2_*_)_-*SNAP^Cd^* | Kint *et al.*, 2020 |  |
| pDIA6458 | pFT47-P_(_*_fdpF_*_)_-*SNAP^Cd^* | Kint *et al.*, 2020 |  |
| pDIA7285 | pFT47-P_σA(_*_revrbr2_*_)_-*SNAP^Cd^* | This work |  |
| pDIA7290 | pFT47-P_σB(_*_revrbr2_*_)_-*SNAP^Cd^* | This work |  |

*erm*: erythromycin resistance cassette

**Table S2. List of oligos used in this study**

| Name | Sequence |  |
| --- | --- | --- |
| OS211 | AAAAAAGCTTATAATTATCCTTAAACAACGATATGGTGCGCCCAGATAGGGTG | IBS-rex-193s |
| OS212 | CAGATTGTACAAATGTGGTGATAACAGATAAGTCGATATGGTTAACTTACCTTTCTTTGT | EBS1d-rex-193s |
| OS213 | TGAACGCAAGTTTCTAATTTCGGTTTTGTTCCGATAGAGGAAAGTGTCT | EBS2-rex-193s |
| OS218 | AAGAAGGCTCCCAAAATATCA | *rex* 5' intron |
| OS219 | GGTGCAAAATTCCATACACCT | *rex* 5' intron |
| EBSU | CGAAATTAGAAACTTGCGTTCAGTAAAC |  |
| CM1 | TGGTCATGAGATTATCAAAAGGAGATAAAGTTACAATATTATAGGAGGAGGT | ACE *CD1777* |
| CM2 | TTTTTTAACAGTAGAGCATTTTGTATATCC | ACE *CD1777* |
| CM3 | TACAAAATGCTCTACTGTTAAAAAATGGAAAAGTACTTTGCTAAAAGAAGAT | ACE *CD1777* |
| CM4 | ATCGTAGAAATACGGTGTTTTTTAGCTATATGTTCTTCATCTCTAGGAC | ACE *CD1777* |
| CM5 | TTGGTTATAGTATACAATTTATGGGTA |  |
| CM6 | CTATAAATATAGAGGCAATTTACAAAA |  |
| IMV694 | GACCCAATTCTCTTATATTTCATACCA | 5’RACE *CD1777* |
| IMV753 | TTTACTAGGAGGGTTTTGAACCA | 5’RACE *CD1777* |
| IMV1211 | CTAAATTATTTTCTTAAGCCAATT | 5’RACE *CD1777* |
| IMV1576 | CAACCAAGTTTTGATGTCAAAT | INV PCR P_σB(_*_revrbr2_*_)_ |
| IMV1577 | GACTTTATAGCTTAAACACTAAAA | INV PCR P_σA(_*_revrbr2_*_)_ |
| IMV1578 | TTAAGCTATAAAGTCCTTGTAGCAAGTTATTAAACTTAAA | INV PCR P_σB(_*_revrbr2_*_)_ |
| IMV1588 | CATCAAAACTTGGTTGTTAGGAAATAAATTTTATTTTTAGTGT | INV PCR P_σA(_*_revrbr2_*_)_ |
| QRTBD3 | TTTTGTTGTGTCTATGAACCTTTGT | RT-qPCR *gyrA* |
| QRTBD4 | TCCTTTACCAGCTCTTATTTGACTT | RT-qPCR *gyrA* |
| QRTBD43 | CAGATGATGTAGGTGGTCGTTTTT | RT-qPCR *pgi* |
| QRTBD44 | AGCAGCAATAGGAAGTAACCCAAC | RT-qPCR *pgi* |
| IMV575 | CCAAGGCAAGATAGCAGGAG | RT-qPCR *fdpA* |
| IMV576 | CTGCACCAAATGCCATACAC | RT-qPCR *fdpA* |
| IMV668 | TGCCCTGTATGTGGAGCTAA | RT-qPCR *fdpF* |
| IMV669 | CCAGCTGCTCCATTTCCTAC | RT-qPCR *fdpF* |
| IMV670 | ACATGAAGGAGATGCTGCAC | RT-qPCR *revrbr1*/2 |
| IMV671 | GTGCTCATCAGCCCAATTTT | RT-qPCR *revrbr1* |
| IMV672 | ATGTTCATCAGCCCAAACCA | RT-qPCR *revrbr2* |
| IMV694 | GACCCAATTCTCTATATTTCATACCA | RT-qPCR *CD1777* |
| IMV693 | TGGTTCAAAACCCTCCTAGTAAA | RT-qPCR *CD1777* |
| QRTBD155 | AGAGAAAGACGAAGCATTTGGTATG | RT-qPCR *adhE* |
| QRTBD156 | GCAGTAGAAGTTGGATTAGTTGTAGG | RT-qPCR *adhE* |
| JP495 | ACATGCTTCAGACTCAAGGTTT | RT-qPCR *grdE* |
| JP496 | CCACTTACAATTGCTCCGTCC | RT-qPCR *grdE* |
